# *TrpA1* Regulates Defecation of Food-Borne Pathogens under the Control of the Duox Pathway

**DOI:** 10.1371/journal.pgen.1005773

**Published:** 2016-01-04

**Authors:** Eun Jo Du, Tae Jung Ahn, Ilmin Kwon, Ji Hye Lee, Jeong-Ho Park, Sun Hwa Park, Tong Mook Kang, Hana Cho, Tae Jin Kim, Hyung-Wook Kim, Youngsoo Jun, Hee Jae Lee, Young Sik Lee, Jae Young Kwon, KyeongJin Kang

**Affiliations:** 1 Samsung Biomedical Research Institute, Sungkyunkwan University School of Medicine, Suwon, Korea; 2 Department of Anatomy and Cell Biology, Sungkyunkwan University School of Medicine, Suwon, Korea; 3 Department of Biological Sciences, Sungkyunkwan University, Suwon, Korea; 4 Department of Physiology, Sungkyunkwan University School of Medicine, Suwon, Korea; 5 Department of Immunobiology, Sungkyunkwan University School of Medicine, Suwon, Korea; 6 College of Life Sciences, Sejong University, Seoul, Korea; 7 School of Life Sciences, Gwangju Institute of Science and Technology (GIST), Gwangju, Korea; 8 Kangwon National University College of Medicine, Chuncheon, Korea; 9 College of Life Sciences and Biotechnology, Korea University, Seoul, Korea; Imperial College London (MRC Clinical Sciences Centre), UNITED KINGDOM

## Abstract

Pathogen expulsion from the gut is an important defense strategy against infection, but little is known about how interaction between the intestinal microbiome and host immunity modulates defecation. In *Drosophila melanogaster*, dual oxidase (Duox) kills pathogenic microbes by generating the microbicidal reactive oxygen species (ROS), hypochlorous acid (HOCl) in response to bacterially excreted uracil. The physiological function of enzymatically generated HOCl in the gut is, however, unknown aside from its anti-microbial activity. *Drosophila* TRPA1 is an evolutionarily conserved receptor for reactive chemicals like HOCl, but a role for this molecule in mediating responses to gut microbial content has not been described. Here we identify a molecular mechanism through which bacteria-produced uracil facilitates pathogen-clearing defecation. Ingestion of uracil increases defecation frequency, requiring the *Duox* pathway and *TrpA1*. The *TrpA1(A)* transcript spliced with exon10b (*TrpA1(A)10b*) that is present in a subset of midgut enteroendocrine cells (EECs) is critical for uracil-dependent defecation. TRPA1(A)10b heterologously expressed in *Xenopus* oocytes is an excellent HOCl receptor characterized with elevated sensitivity and fast activation kinetics of macroscopic HOCl-evoked currents compared to those of the alternative TRPA1(A)10a isoform. Consistent with *TrpA1*’s role in defecation, uracil-excreting *Erwinia carotovora* showed higher persistence in *TrpA1-*deficient guts. Taken together, our results propose that the uracil/Duox pathway promotes bacteria expulsion from the gut through the HOCl-sensitive receptor, TRPA1(A)10b, thereby minimizing the chances that bacteria adapt to survive host defense systems.

## Author Summary

The amount and pattern of defecation are often determined by the bacterial composition in the gut, and can have a significant impact on human health. It is however unknown how changes in the bacterial community affect defecation. A chemical defense system called the *Duox* pathway is known to kill ill-causing bacteria in the gut by producing chlorine bleach. Our study reveals that the bleach does not stop there, working further to promote expulsion of the bacteria from the gut through a sensitive bleach detector. Moreover, in the gut deficient for the bleach detector, the bacteria not only stayed longer, but also opportunistically survived the bleach that they encounter afterwards. This is the first identification of a mechanism illustrating how important it is that gut defense systems control defecation, and helps explain why troubles in the gut cause changes in defecation.

## Introduction

Encountering other organisms in nature offers opportunities of benefits or dangers depending on the relationship of the organisms facing each other. We intimately interact with bacteria in the gut, and the control of such interaction is the key to the health of animals [1–3]. Although pathogen expulsion from the gut is an important defense measure to infection [4], it is not clearly determined how defecation contributes to the defense against food-borne bacterial pathogens in collaboration with innate immune systems in the intestine. Bacterial homeostasis in the *Drosophila* gut is under the control of two distinct innate immune mechanisms, the *imd* and *Duox* pathways [5–7]. In the latter, bacteria-originated uracil upregulates Duox activity via G-protein signaling pathways [8–10], which are independent of the *imd* pathway [5,8]. The uracil-stimulated Gαq-PLCβ-Ca^2+^ pathway increases the enzymatic activity of Duox exploiting the intracellular Ca^2+^ -binding domain of Duox [8,9], while the uracil-detecting GPCR has yet to be identified. In addition, the sequential activation of MEKK1-MKK3-p38 MAPK, which depends on PLCβ, upregulates transcription of the *Duox* gene [10]. The upregulation of *Duox* dramatically increases the concentration of highly microbicidal HOCl [8,11] in the gut lumen, as the ROS is enzymatically generated by collaboration of the two cytosolic and extracellular oxidase domains of Duox [12]. The intracellular gp91^*phox*^-like oxidase domain of Duox extracts electrons from NADPH, and the electrons are delivered to the extracellular space through two heme structures in the transmembrane domains of Duox, sequentially forming superoxide and hydrogen peroxide (H_2_O_2_). The extracellularly generated H_2_O_2_ is then subjected to peroxidation by the peroxidase homology domain (PHD) of Duox to generate microbicidal HOCl. Thus, the Duox pathway plays a critical role for the control of the gut microbiome homeostasis by responding to bacterially excreted uracil and producing the reactive chlorine oxidant, HOCl.

Gastrointestinal motility is a key determinant of defecation in mammals, and is controlled by enteroendocrine cells as well as enteric and central nervous systems. The majority of endocrine cells in the small and large intestine consists of enterochromaffin cells (EC cells) which contain >90% of intestinal serotonin. Serotonin in EC cells is closely associated with gastrointestinal motility [13,14]. Recently, the EC cells are reported to express TRPA1, and the activation of TRPA1 contributes to the gut motility through serotonin release [13] albeit with no known indigenous signal inputs capable of stimulating TRPA1 activity in EC cells. Insect intestinal motility shows similar operational principles, as gut motility of *Drosophila* larvae was reported to be modulated by diuretic hormone 31 (DH31) [15] which is released from subsets of enteroendocrine cells [16,17]. Thus, *Drosophila* provide the intestinal model system to study functional implication of the enteroendocrine system in various contexts including host/microbe interaction in the intestine.

As a reactive chemical, HOCl activates the mammalian ortholog of the evolutionarily conserved reactive chemical receptor TRPA1 [18]. TRPA1s from humans to flies share the mechanism through which the channels are activated by reactive chemicals [19]. While insect TRPA1s are key receptors for both temperature [20,21] and reactive chemicals, it was shown that sensory discrimination between these two sensory cues is achieved by expressing a thermally insensitive TRPA1 isoform, TRPA1(A), in taste neurons [19,20,22]. In addition to lacking high thermal sensitivity, fruitfly and mosquito TRPA1(A)s have recently been reported to respond to the phytochemical citronellal in an isoform-specific manner [23], suggesting that TRPA1(A) is a chemosensory-specialized isoform. In addition, a small domain between the N-terminal ankyrin repeats and the transmembrane segment is alternatively encoded by exon10a and exon10b in *Drosophila melanogaster* [24], which was proposed to be another determinant of thermal sensitivity [25]. However, the physiological significance of the transcript with exon10b has not been fully established.

Recently two independent transcriptome analyses showed that *TrpA1* transcripts are present in the *Drosophila* gut [26,27], but the role of *Drosophila TrpA1* in the gut has yet to be examined. In this study, we find that the TRPA1(A) isoform coupled with exon10b (referred to as TRPA1(A)10b) is the key receptor for the detection of the major reactive chlorine HOCl generated from activation of the *Duox* pathway in the gut and that such functional interaction between TRPA1 and the Duox pathway is critical for expulsion of the opportunistic pathogen *Erwinia carotovora* from the gut. These results indicate that the Duox innate immune system regulates defecation via TRPA1, thus maintaining the microbiome homeostasis in the *Drosophila* gut.

## Results and Discussion

### Orally ingested uracil increases defecation frequency through *TrpA1*

**Fig 1:**
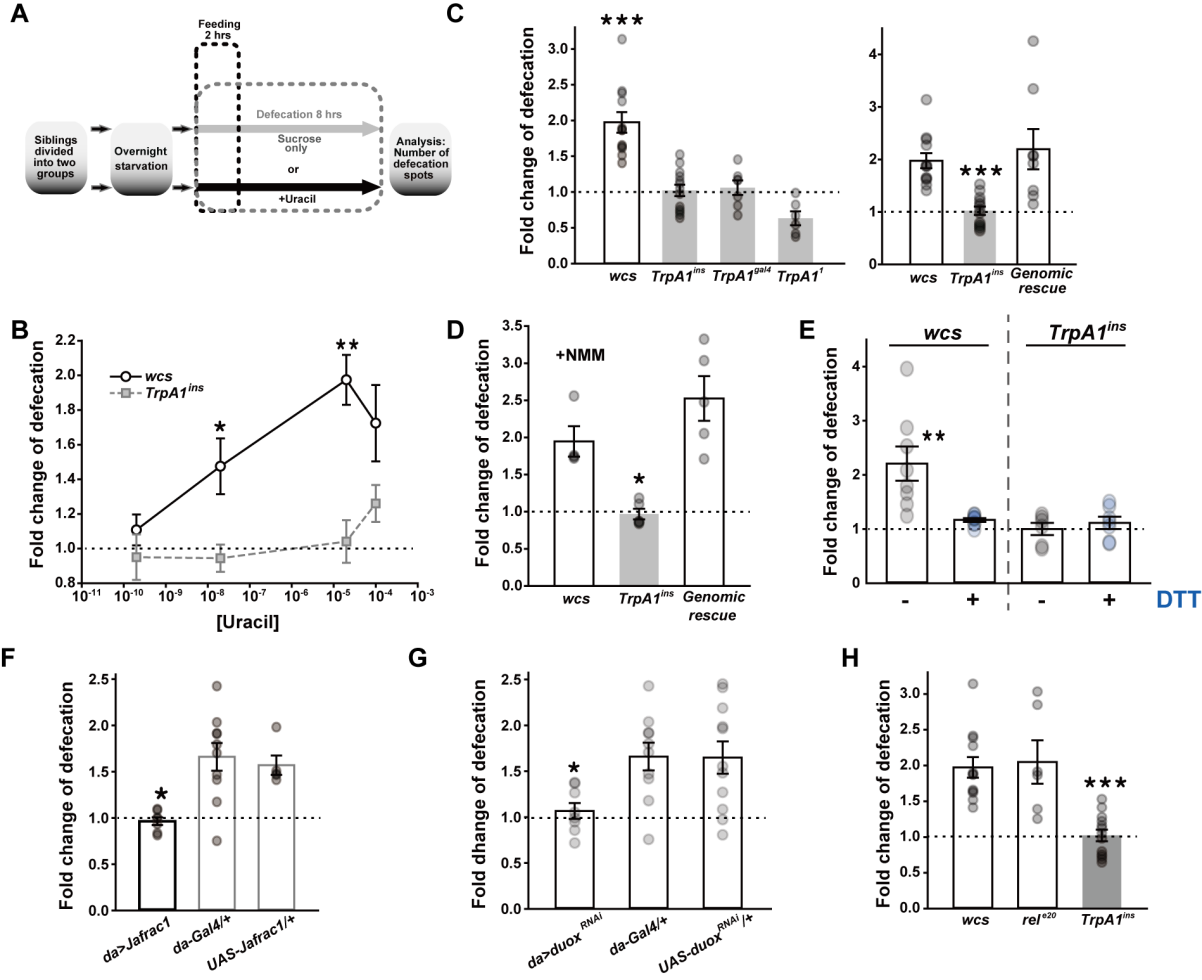
TRPA1 regulates defecation in response to the uracil-triggered Duox pathway. **A** Schematic diagram illustrating the uracil-dependent defecation assay (see Materials and Methods for details)**. B** Dose-dependence of uracil-responsive defecation in wild type flies (*wcs*) versus *TrpA1*^*ins*^ (n = 4–13). Sucrose solution containing uracil at 0.2, 20, 20,000 and 100,000 nM was fed to the indicated genotypes, and the defecation was compared with that from sucrose-only-fed flies. **C** Requirement of *TrpA1* for uracil-induced defecation was confirmed with three independent *TrpA1* alleles (n = 6–13). Fold change of defecation was acquired by comparing defecation frequencies from two sibling groups fed with or without 20 μM uracil. *Right*: The defecation defect in *TrpA1*^*ins*^ was rescued by the WT *TrpA1* gene inserted in the fly genome. **D** Ingestion of electrophile N-methyl maleimide (NMM) promotes defecation depending on *TrpA1* (n = 4–5), indicating that reactive chemicals in the gut lumen can induce defecation increases through *TrpA1*. **E** Co-ingestion of ROS-counteracting dithiothreitol (DTT) with uracil abolishes the defecation increase that is dependent on *TrpA1* (n = 6–8). See also S2E–S2G Fig. **F** Ubiquitous expression of a peroxiredoxin gene *Jafrac1* **by *da-Gal4*** readily suppresses uracil-dependent defecation increase (n = 5–10). **G** *Duox* is required for uracil-induced defecation, as RNAi knockdown abolishes uracil-dependent defecation (n = 8–11). **H** The *imd* pathway is dispensable for *TrpA1-*dependent defecation, as animals with the strong loss-of-function allele, *rel*^*e20*^, of the NF- κB gene retain the uracil-dependent defecation response (n = 6–13). Mean ± SEM is shown with data distribution. *: p<0.05, **: p<0.01, ***: p<0.001, Student’s *t*- (**B**) or Tukey tests (**C** to **H**). See also S1 Fig for alternative presentation of data and S3A–S3D Fig for mechanical aspects of uracil in relation to the TRPA1 channel activity and feeding behavior.

To test if the Duox pathway functionally interacts with TRPA1 and regulates defecation, a behavioral assay was designed to compare defecation spot numbers of two sister fly groups fed with solution containing either sucrose only or sucrose and uracil (500 mM sucrose±uracil) (Fig 1A, and Materials and Methods for details). Defecation spot numbers were normalized with respect to ingestion amounts, yielding data sets of either “output/input” (spots/mm) for simple comparison between sucrose only and sucrose+uracil experiments (S1 Fig) or “fold change of defecation” for quantitation of increased defecation by uracil. Increasing concentrations of uracil ranging from 20 nM to 100 μM elevated defecation frequency of wild type flies (*wcs*) (Fig 1B), while three independent alleles, *TrpA1*^*ins*^, *TrpA1*^*Gal4*^ and *TrpA1*^*1*^, which are severely impaired for *TrpA1*-dependent chemical detection [19,28,29], lacked uracil-dependent defecation (Fig 1B and 1C and S1A Fig) as did *TrpA1* RNAi knockdown animals (S1B Fig). Note that such uracil-dependent defecation was not reliably observed with female flies, probably because of physiological complexity of females as observed in previous studies [30,31]. For this reason, only male flies were tested for all experiments. Reintroduction of the *TrpA1* gene (genomic rescue [20]) restored uracil-dependent defecation in *TrpA1*^*ins*^ animals (Fig 1C and S1A Fig). To examine if the number of fecal spots represents the approximate amount of defecation, the vial, in which flies were let feed on 0.5% brilliant blue FCF-containing 500 mM sucrose solution and defecate for 8 hrs in total, was washed inside with 2.5 ml phosphate-buffered saline (PBS) (S2C Fig). Spectral absorbance at 628 nm was determined for quantitation of the blue dye, and the results were very similar to those appraised by counting fecal spots. Uracil did not significantly affect gustatory behavior to sucrose (S3D Fig) or ingestion of sucrose solutions (S1F Fig). Application of 20 μM extracellular uracil did not activate *Drosophila* TRPA1(A)10a or TRPA1(A)10b expressed in *Xenopus* oocytes (S3A–S3C Fig), indicating that uracil does not directly stimulate TRPA1. Thus, ingested uracil increases defecation likely by activating a signaling pathway upstream of *TrpA1*.

### Reactivity of ROS resulting from uracil-dependent Duox activation increases defecation

To examine whether the reactivity of ROS resulting from *Duox* activation is important for *TrpA1*-dependent defecation, N-methylmaleimide (NMM), a robust sulfhydryl-reactive chemical which activates TRPA1 [19,22,32], was fed at 1 mM replacing uracil. The TRPA1 agonist increased defecation frequency in *wcs* with the extent similar to that of 20 μM uracil with *TrpA1* required (Fig 1D and S1C Fig). However, NMM ingestion did not elevate HOCl production when the guts from NMM-ingested animals were examined with the HOCl-specific fluorescent dye, R19S [8,33] (S4B–S4E Fig). This result may suggest that *Duox*-derived chemical reactivity is crucial for *TrpA1*-dependent defecation. Indeed, the ROS-counteracting dithiothreitol (DTT, 10 mM) fed with uracil abolished uracil-dependent defecation increase in *wcs* without significantly affecting defecation of a *TrpA1* mutant (Fig 1E) or the gustatory behavior (S3D Fig). Co-ingestion of uracil and DTT is unlikely to change their chemical properties, since two hour incubation of the mixture at room temperature did not alter their spectral absorbances (S3E–S3G Fig). With the use of R19S, it was also confirmed that the HOCl level was readily reduced by co-ingestion of DTT with uracil (S4B–S4E Fig). Consistently, overexpression of the peroxiredoxin gene *Jafrac1* [34] under the control of *da-Gal4* that drives ubiquitous *UAS*–dependent expression and was previously used to RNAi-knockdown *Duox* expression [11] abolished uracil-dependent defecation (Fig 1F). Deterring reactive chemical biosynthesis, RNAi knockdown of *Duox* significantly reduced uracil-elicited defecation (Fig 1G), which confirms the role of Duox in uracil- and *TrpA1*-dependent defecation. Consistent with the biochemical Duox activity observed in dissected guts [11], *Duox* RNAi knockdown in the nervous system by pan-neuronal *appl-Gal4* insignificantly lowered uracil-dependent defecation (S1D Fig), suggesting that HOCl production in the gut is critical for uracil-dependent defecation. In parallel to the reported independent relationship of the other gut innate immune system, the *imd* pathway, with uracil-evoked HOCl production by Duox [6,8], *rel*^*e20*^, a *relish* mutant severely defective for the *imd* immune response [35] showed normal uracil-elicited defecation (Fig 1H). To exclude the possibility that the deficit of defecation caused by genetic manipulation stems from developmental impairment, RNAi knockdown of *Duox* or *TrpA1* and *Jafrac1* overexpression were delayed until one day before defecation experiments by means of *Gal80*^*ts*^-dependent transcriptional suppression of *Gal4* (S5 Fig), which produced outcomes similar to corresponding experiments described above. Taken together, the requirement of *Duox* expression and chemical reactivity of its microbicidal ROS product point out that the TRPA1 activity is likely under the direct control of Duox, but not cytosolic [Ca^2+^] increase or PIP_2_ depletion by uracil-driven G-protein signaling pathways.

### *TrpA1* expressed in a subset of enteroendocrine cells is necessary for uracil-evoked defecation increase

**Fig 2:**
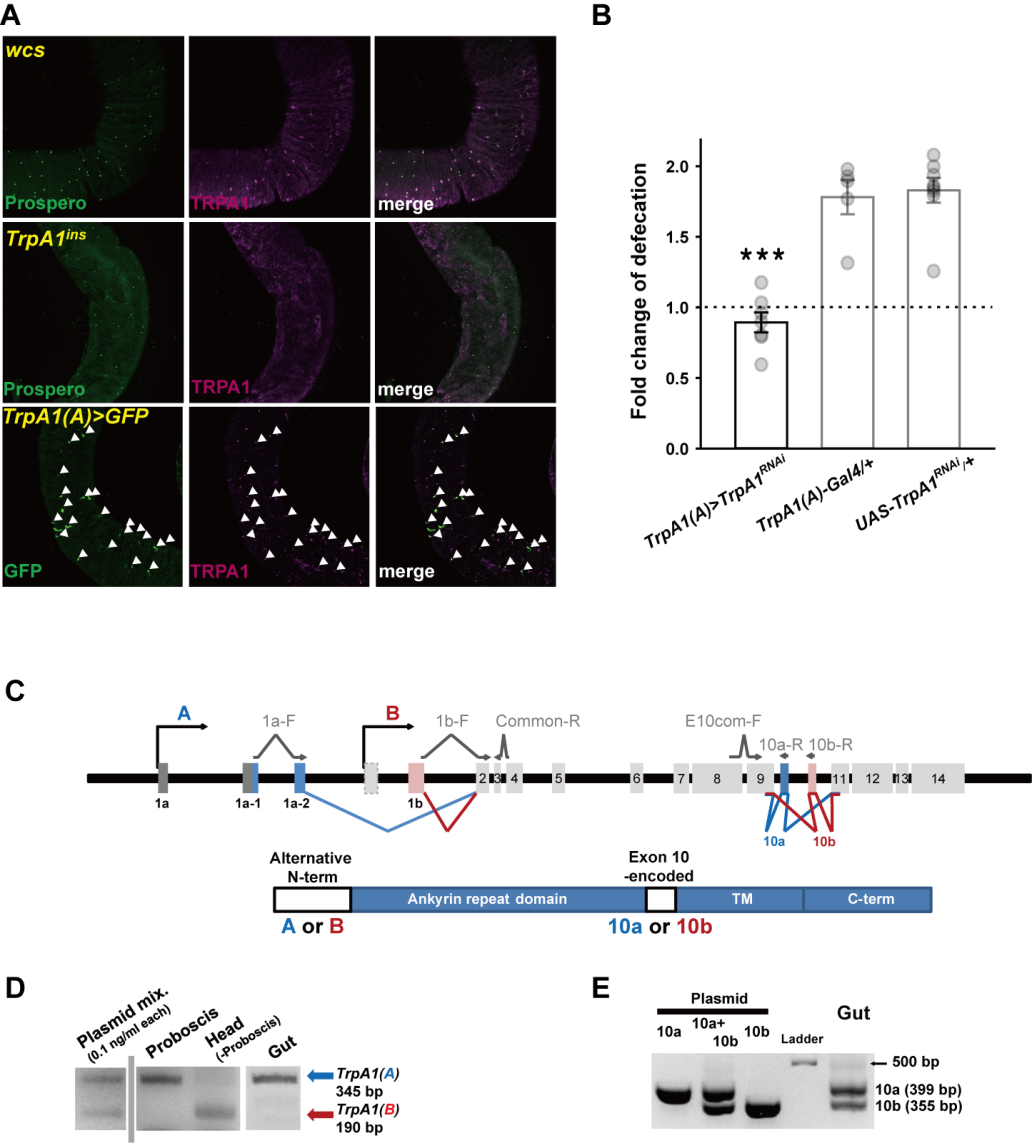
*TrpA1(A)* in a subset of enteroendocrine cells is necessary for uracil-dependent defecation. **A** Co-immunostaining of TRPA1 and either Prospero or GFP in the anterior midgut from indicated genotypes. *Bottom*, Arrowheads: TRPA1 and GFP co-immunostaining. **B** *TrpA1* RNAi in *TrpA1(A)-Gal4* EECs dramatically suppresses uracil-dependent defecation (n = 5–8). **C** *TrpA1* gene structure illustrating alternative exon usage (*Top*) and TRPA1 protein organization showing variable domains (*Bottom*). The primers used in reverse transcription PCR analyses shown in (**D** and **E**) are indicated as arrows. Most of primers in these analyses were designed to straddle two separate exons in order to prevent PCR amplification of genomic DNA. **D** and **E** The representative results of RT-PCR analyzing alternative exons of *TrpA1* transcripts in the gut for the N-terminal (**D**) and exon10-encoded domains (**E**). ‘Plasmid’ indicates the PCR reactions with cloned cDNAs of *TrpA1* transcripts as DNA polymerization templates, and the bands resulting from the‘plasmid’ reaction were used as size and intensity markers. Proboscis, Head and Gut denote the tissues where total RNA was prepared for reverse transcription. The band of the “Ladder” lane is a 500 bp long DNA fragment of Generuler 1 kb ladder (**E**). 10a and 10b indicate cDNA templates or PCR products of *TrpA1(A)10a* and *TrpA1(A)10b*, respectively, and the PCR products from the template of cloned cDNA (indicated by ‘Plasmid’) serve as size markers for the RT-PCR experiments with total cDNA prepared from the gut. Bar graphs show mean ± SEM with distribution. ***: p<0.001, Tukey test.

**Fig 3:**
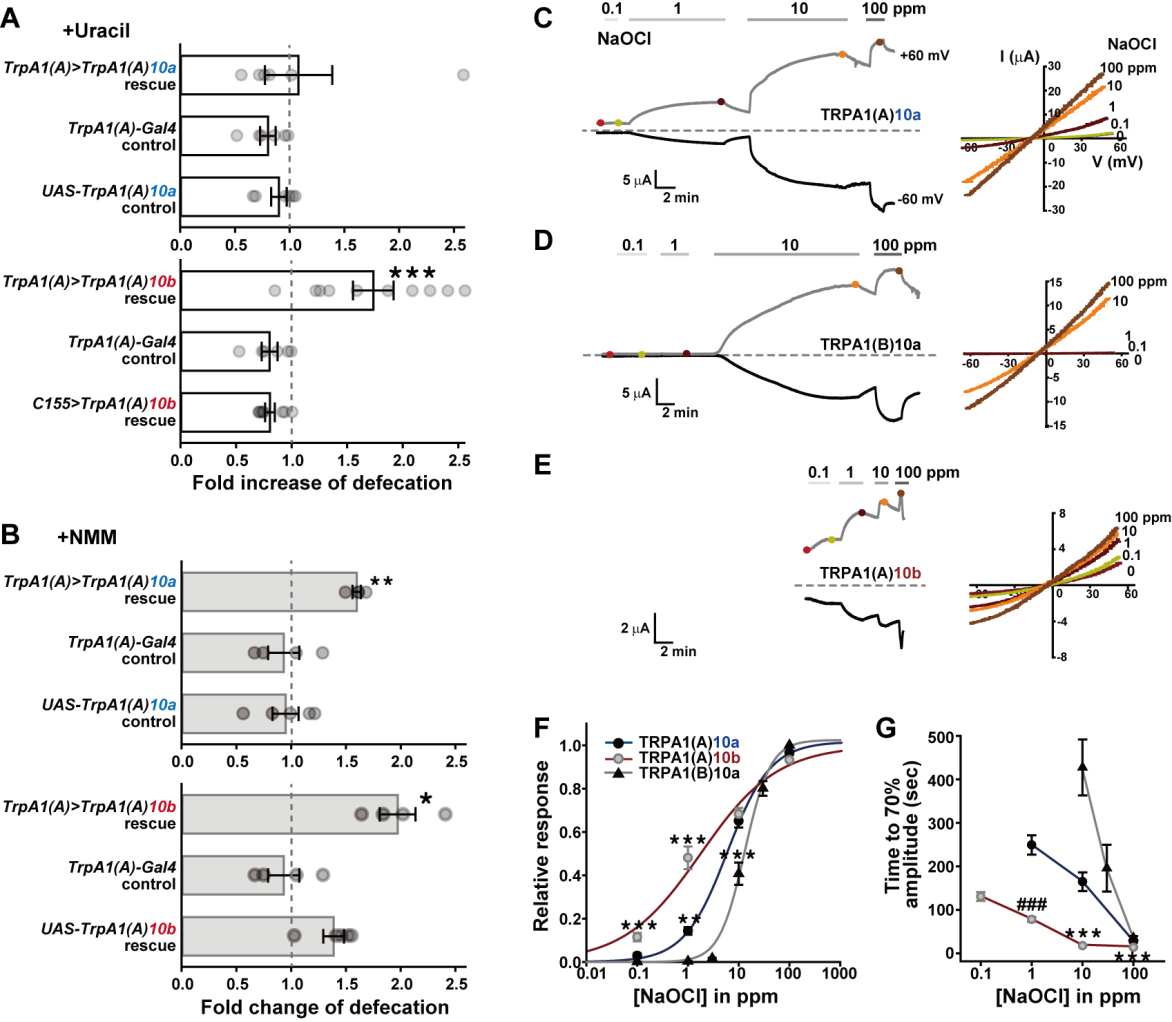
The TRPA1(A)10b isoform is the HOCl receptor essential for uracil-evoked defecation. **A** Rescue of the defect in uracil-dependent defecation of *TrpA1*^*ins*^ animals by expressing *TrpA1(A)* cDNA with either exon10a (*TrpA1(A)10a*) or exon10b (*TrpA1(A)10b*) in *TrpA1(A)-Gal4* cells (n = 6–10). Expression of *TrpA1(A)10b* by EEC-expressing *TrpA1(A)-Gal4* but not pan-neuronal *c155-Gal4* restored uracil-dependent defecation in the *TrpA1*^*ins*^ animal (*Lower*), while *TrpA1(A)10a* expression in *TrpA1(A)-Gal4* EECs did not (*Upper*). **B** Rescue of the defect in NMM-dependent defecation of *TrpA1*^*ins*^ mutants by the two alternative *TrpA1(A)* isoforms (n = 4–5). The NMM-mediated defecation was rescued by expressing TRPA1(A)10a in *TrpA1(A)-Gal4* EEC cells, although uracil-dependent defecation was not. **C-E** Representative dose dependence (*Left*) and current-voltage relationship (*Right*) of NaOCl responses in *Xenopus* oocytes heterologously expressing indicated isoforms. Note that the time scales and dose sequences are set identical for simple comparison of activation kinetics. **F** Normalized dose dependences of TRPA1 isoforms averaged from the results at -60 mV in **C**-**E** panels (n = 4–10). TRPA1(A)10b show more sensitive response to NaOCl than TRPA1(A)10a or TRPA1(B). Current amplitudes at varying doses of NaOCl was normalized with respect to that at 100 ppm. The data were fitted to Hill equation to determine EC50s. **G** Time to 70% of the steady state current amplitude induced at each NaOCl concentration was measured for indicated TRPA1 isoforms for activation kinetics comparison of macroscopic NaOCl currents (n = 5–9, -60 mV). Mean ± SEM shown. *: p<0.05, **: p<0.01, ***: p<0.001, Tukey test. ###: p<0.001, Student’s *t*-test. See also S9 Fig for further functional characterization data of fly and human TRPA1s in oocytes, and for HOCl- or NMM-elicited action potential responses in sugar cells ectopically expressing fly TRPA1 isoforms.

Highly reactive HOCl produced by Duox was previously shown to accumulate in the lumen of the gut [8] where *TrpA1* transcripts appear to be present [26,27]. Indeed, the fly midgut showed TRPA1 immunoreactivity which was absent in *TrpA1*^*ins*^ (Fig 2A, *Middle* and S6 Fig for higher magnification). The immunoreactivity was observed across most of the anterior midgut and in two lateral domains across middle midgut boundaries facing the anterior or posterior midgut (illustrated in S7A Fig). Interestingly, the TRPA1-expressing cells were closely associated with immunoreactivity of the enteroendocrine cell (EEC) nuclear marker, Prospero (Fig 2A, *Top*) [36], but not with those of intestinal stem cell and enteroblast markers (S8A and S8B Fig). Note that the background staining that sometimes appears in the visceral muscle is non-specific or not critical for the defecation, because it was sometimes observed in the *TrpA1*-deficient guts and *TrpA1* RNAi-knockdown by EEC-restricted *TrpA1(A)-Gal4* phenocopies *TrpA1*^*ins*^. *TrpA1(A)*-*Gal4* contains the genomic fragment of the *TrpA1(A)* isoform-specific promoter upstream of the *Gal4* coding sequence [25]. GFP expression driven by *TrpA1(A)*-*Gal4* comprised a subset of Prospero-positive cells (S7B Fig) as in TRPA1 immunostaining, and was colocalized with the majority of anti-TRPA1 cells (Fig 2A, *Bottom*) throughout the anti-TRPA1-stained regions. In order to functionally demonstrate the role of *TrpA1*-positive cells in the defecation, *TrpA1* RNAi knockdown was conducted with *TrpA1(A)-Gal4*, and showed significant reduction of TRPA1 expression in EECs (S8C Fig) and uracil-dependent defecation (Fig 2B and S7C Fig). On the other hand, pan-neuronal expression of *TrpA1* cDNA by *c155-Gal4* was unable to rescue the defecation defect in *TrpA1*^*ins*^ mutants, whereas that by EEC-covering *TrpA1(A)-Gal4* did (Fig 3A, *Lower*). These observations together with the *Duox*-dependent HOCl production in the gut lumen [8,11] suggest that the known peripheral and central *TrpA1-*positive neurons so far identified by immunostaining [19,20,22,37] are not primarily related to uracil-elicited defecation, advocating the role of *TrpA1*-expressing EECs in uracil-dependent defecation.

### The TRPA1(A)10b isoform expressed in the gut EECs is the key HOCl receptor

In sensory systems, it has been reported that alternative use of exons (Fig 2C) allows *TrpA1* to conform to multiple sensory needs by modulating its temperature sensitivity [22,25]. Our reverse transcription analyses of dissected guts revealed that *TrpA1(A)*, the chemosensory isoform expressed in taste neurons with much reduced thermal sensitivity [22], is predominant in the gut (Fig 2D) and can be alternatively spliced with either exon10a or exon10b (Fig 2E). The exon10 encodes a small region consisting of 37 or 36 amino acids of exon10a or exon10b, respectively, between the N-terminal ankyrin repeat and transmembrane domains (Fig 2C, *Lower)*. Interestingly, *TrpA1(A)10b* but not *TrpA1(A)10a* cDNA restored the uracil-dependent defecation when expressed in *TrpA1(A)-Gal4* cells of *TrpA1*^*ins*^ mutants (Fig 3*A*). The ineptitude of the *UAS-TrpA1(A)10a* transgene in restoring the defecation is unlikely due to lack of functional expression based on two lines of evidence. First, functional expression of *UAS-TrpA1(A)10a* was comparable to that of *UAS-TrpA1(A)10b* in gustatory neurons (S9H and S9I Fig). Second, defecation increase caused by ingestion of the TRPA1 agonist NMM was restored in the *TrpA1* mutants by expressing either *TrpA1(A)10a* or *TrpA1(A)10b* in the *TrpA1(A)-Gal4* cells (Fig 3B), as was in the genomic rescue animals (Fig 1D). TRPA1(A)10a and TRPA1(A)10b share the key cysteines in the N-terminal ankyrin repeat domain, which are important for detection of electrophilic chemicals [19,32,38], and both the isoforms should thus be able to respond to NMM. The defecation increase of *TrpA1(A)10a-*expressing *TrpA1*–deficient animals in response to NMM ingestion demonstrates the functional expression of the *UAS-TrpA1(A)10a* transgene in the *TrpA1(A)-Gal4* cells where expression of *TrpA1(A)10a* was unable to restore uracil-evoked defecation in *TrpA1*-deficient animals, while the former result (S9H and S9I Fig) indicates successful transgenesis of *UAS-TrpA1(A)10a*. The isoform dependence in uracil-evoked defecation might result from further functional diversification of TRPA1(A) via alternative exon10s altering response parameters to HOCl of the channel isoforms. To examine for any differential HOCl responsiveness of the two isoforms, TRPA1 isoforms were heterologously expressed in *Xenopus laevis* oocytes, and HOCl responses of the ion channel isoforms were characterized by the two-electrode voltage clamping approach. Interestingly, TRPA1(A)10b showed >3 times faster activation kinetics at 1 and 10 ppm of the HOCl-donating NaOCl at the membrane potential of -60 mV (Fig 3C–3E and 3G) and >3 times higher responsiveness to 0.1 and 1 ppm NaOCl at -60 mV (Fig 3F) than other fly TRPA1 variants and human TRPA1 (S9A–S9F Fig). Furthermore, in contrast to comparable responses of the two fly TRPA1 variants to NMM in gustatory neurons, *Gr5a-Gal4* chemosensory neurons ectopically expressing *TrpA1(A)10b*, but not those expressing *TrpA1(A)10a*, showed spiking frequency increases in response to NaOCl 100 ppm (S9H and S9I Fig). These data indicate that TRPA1(A)10b expressed in EECs is capable of mediating *Duox*-dependent defecation by rapidly reacting to low amounts of reactive chlorine species that are probably short-lived in the gut mucus due to their high reactivity. The enhanced sensitivity and response kinetics of TRPA1(A)10b might not originate from its intrinsic receptivity to reactive chemicals, because the transcript spliced with Exon10b loses a cysteine that is known to be important for electrophile sensitivity [19,32]. In parallel with this view, the recently characterized non-covalent TRPA1(A) agonist citronellal [23] activates TRPA1(A)10b with higher sensitivity and response kinetics than TRPA1(A)10a (S10 Fig) as does NaOCl, suggesting that the enhanced NaOCl receptivity of TRPA1(A)10b is not because it responds better to chemical reactivity than TRPA1(A)10a.

### *TrpA1* is required for defecation increase by uracil-producing *Erwinia carotovora* subspecies *carotovora* 15

**Fig 4:**
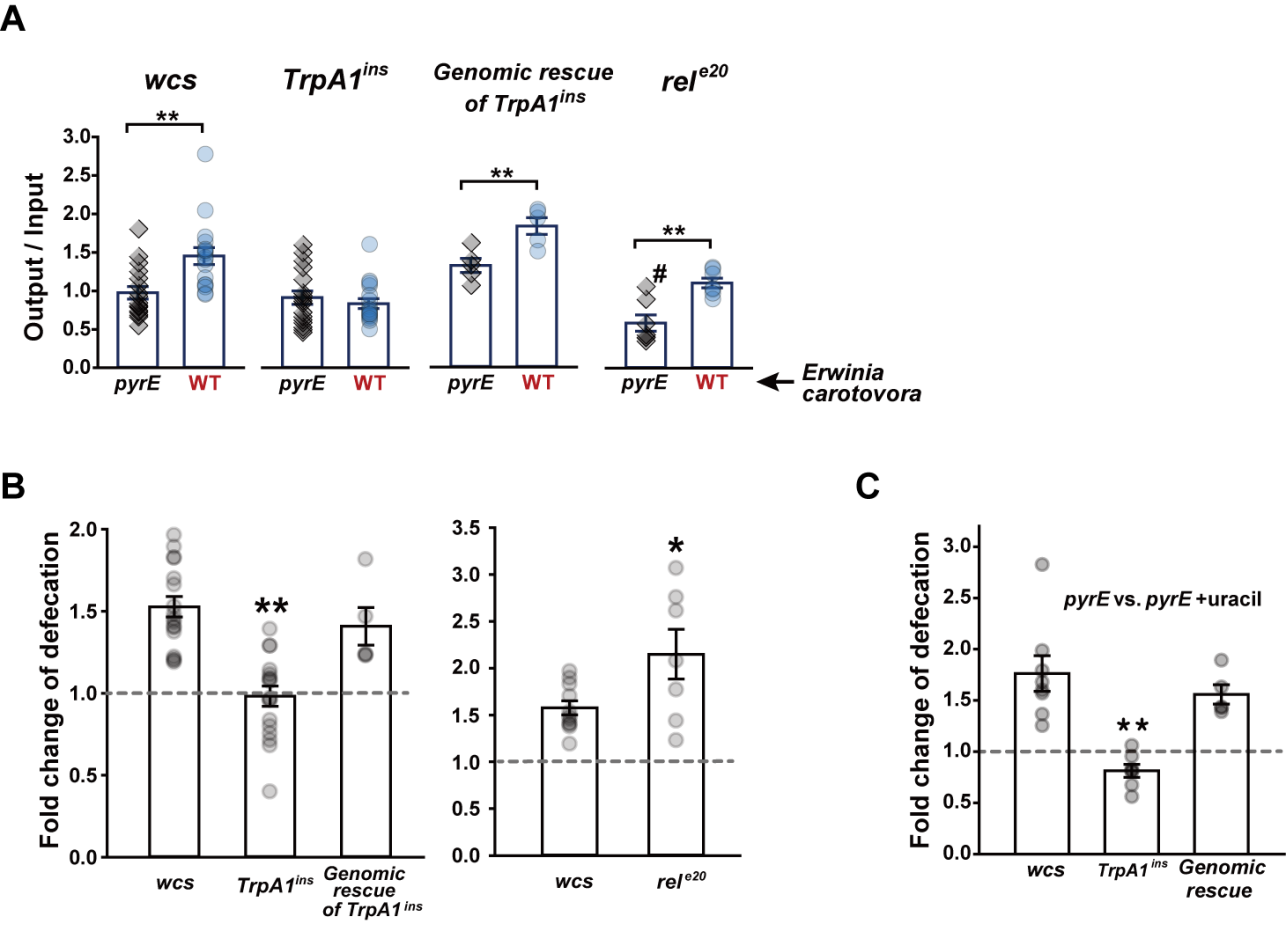
*TrpA1*-dependent defecation is required for bacteria expulsion from the gut. **A** Defecation of indicated *Drosophila* lines fed with either Ecc15 *pyrE* or Ecc15 wild-type (WT) was presented as output/input denoting the fecal spot number normalized by ingestion volume (n = 5–17). **B** Quantitative representation of (A) as defecation fold difference of indicated genotypes. *Left*, The *wcs* flies fed with ECC15 WT at OD600 of 10 defecate ~1.5 fold more than those fed with ECC15 *pyrE*, while *TrpA1*^*ins*^ did not show higher defecation with ECC15 WT. *Right*, *rel*^*e20*^ lacking the *imd* pathway show higher defecation frequency with ECC15 WT over *pyrE*. **C** Defecation comparison between *pyrE* ingestions with and without supplementation of 1 mM uracil (n = 5–8). Defecation of ECC15 *pyrE-*ingested flies is increased by supplementing uracil, and the increase requires *TrpA1*. #: p<0.05, Student’s *t-*test between *wcs* and *rel*^*e20*^ fed with Ecc15 *pyrE* (**A**). *: p<0.05, **: p<0.01, Student’s *t-* or Tukey tests.

*Erwinia carotovora* subspecies *carotovora* 15 (Ecc15), the well-characterized microbe excreting uracil, is sensitive to the Duox immune response [8,11]. The uracil-deficient mutant strain, Ecc15 *pyrE*, was incapable of eliciting reactive chlorine production in the fly gut [8]. Similar to the nonbacterial uracil experiments above, oral ingestion of uracil-producing Ecc15 WT induced higher defecation frequencies than that of uracil-lacking Ecc15 *pyrE*, a trait requiring *TrpA1* but independent of the *imd* pathway in flies (Fig 4A and 4B).Ingestion of the bacteria raises overall levels of defecation compared to sucrose conditions. WT flies consuming *pyrE* or WT ECC15 showed defecation spots per ingested volume (output/input) of 0.98+/-0.08 or 1.45+/-0.11, while ingestion of sucrose only or sucrose+uracil was led to output/input of 0.41+/-0.04 or 0.77+/-0.05 (S1 Fig), respectively. The defecation increase by bacterial ingestion was similarly observed in the *TrpA1*-deficient flies. This result is in contrast to the previous observation where ECC15 ingestion did not alter defecation patterns compared to ingestion of LB+sucrose [30]. We suspect that this discrepancy might have resulted from difference in experimental conditions, as our experiments with sucrose ingestion did not include the LB medium offering protein. The difference in defecation between the two bacterial strains is unlikely due to general physiological incompetence of Ecc15 *pyrE*, as Ecc15 *pyrE* ingestion supplemented with 1 mM uracil led flies to defecate more than unsupplemented ingestion, which again requires *TrpA1* (Fig 4C).

### ECC15 persist longer in the fly guts deficient of *TrpA1* and opportunistically acquire transient HOCl resistance

**Fig 5:**
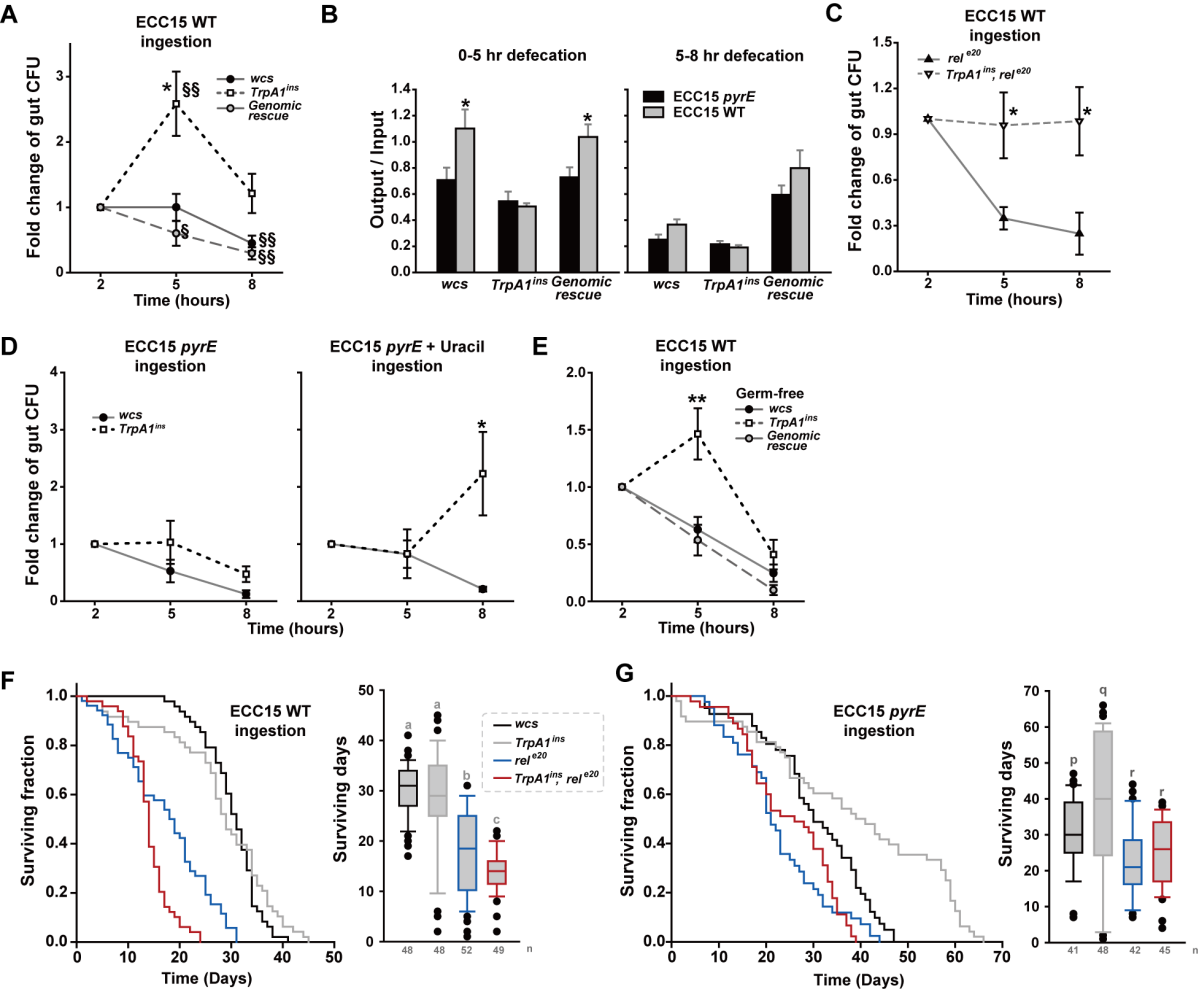
Defecation deficit in *TrpA1* mutants is associated with longer ECC15 gut persistence and *rel-*dependent increased mortality. **A** Gut persistence presented as relative colony-forming units (CFUs) at 2-, 5- and 8-hr time points of the experiments (n = 6–12). The time point indicates the time after start of ECC15 feeding. After ingestion of ECC15-GFP for indicated time, the dissected intestine was homogenized and CFUs of intestinal ECC15-GFP were determined to estimate bacterial growth in the gut. CFUs at 5 and 8 hr time points were divided by that at 2 hr time point to minimize experimental variation. **B** Defecation in the assay time windows of 0–5 hrs and 5–8 hrs is displayed as output/input (n = 5–17). Consistent with (**A**), *TrpA1*-dependent defecation is more prominent between 0 and 5 hr time points. **C** Loss of *TrpA1* increases ECC15 gut persistence in the gut deficient for the *imd* pathway. **D** Ingestion of ECC15 *pyrE* does not differentiate *wcs* and *TrpA1*^*ins*^ in gut persistence (*Left*). Supplementation with uracil allows ECC15 *pyrE* to proliferate in the guts of *TrpA1*^*ins*^ more than *wcs* albeit at a delayed time point of 8 hrs (*Right*). **E** Germ-free flies show similar difference in ECC15 gut persistence among the *wcs*, *TrpA1*^*ins*^ and genomic rescue strains, indicating that the persistence phenotypes resulted from genetic manipulation in flies not commensal bacteria which may differ in the fly strains. **F** and **G** Ingestion of ECC15 shortens life span of immune-compromised flies. When daily ingesting ECC15 WT (**F**), the *rel*^*e20*^ mutant defective in the *imd* innate immune pathway shows reduced life span, which was further exacerbated by additional loss of *TrpA1*, indicating uracil-dependent defecation contributes to ECC15 resistance. Ingestion of ECC15 *pyrE* did not differentiate *rel*^*e20*^ and *TrpA1*^*ins*^, *rel*^*e20*^. *Left*, Kaplan-Meier LogRank survival analysis of indicated genotypes ingesting ECC15 WT (**F**) or *pyrE* (**G**) (p<0.001). *Right*, Life span distribution of the animals tested in the panel of *Left*. All pairwise multiple comparison by Holm-Sidak method. Letters indicate significantly distinct groups (p<0.001). §: p<0.02, §§: p<0.01, ANOVA Repeated Measures Holm-Sidak method (**A**). Mean ± SEM. *: p<0.05, **: p<0.01, Student’s *t-* or Tukey tests.

Increase of defecation might help flies defend themselves by suppressing growth of gut bacteria. Indeed, the guts from animals lacking *TrpA1* exhibited increased colony-forming units (CFUs) 5 hrs after the start of the 2-hr Ecc15 ingestion session, and CFUs at the 8-hr time point were similar to those after the 2-hr feeding session (Fig 5A). In contrast, CFUs in the guts of *wcs* and genomic rescue animals were similar or lowered, respectively, at the 5-hr time point and were further reduced at 8 hrs, compared to 2 hrs, (Fig 5A). Consistent with this result, 5 hrs after the start of ingestion, defecation was significantly increased with uracil-producing Ecc15 in *TrpA1-*expressing flies (Fig 5B), indicating that uracil-elicited defecation in the experimental time window of 0–5 hrs is responsible for timely pathogen expulsion. These results demonstrate that uracil production in Ecc15 lowers the number of gut Ecc15 by increasing defecation frequencies via TRPA1. Consistent with our results above indicating the *imd* pathway to be independent of uracil- and *TrpA1-*dependent defecation, the gut persistence of ECC15 WT was not elevated rather lowered at the 5- and 8-hr time points in the guts of *rel*^*e20*^(Fig 5C). The *rel*^*e20*^, *TrpA1*^*ins*^ double mutants showed the increased CFU of ECC15 WT in the gut, again pointing out the importance of *TrpA1* in timely defecation of uracil-producing ECC15. Ingestion of ECC15 *pyrE* did not differentiate CFUs from *wcs* and *TrpA1*^*ins*^, which indicates that the growth of the bacterial stain is similar in the guts of *wcs* and *TrpA1*^*ins*^(Fig 5D, *left*). However, inability of uracil excretion was not led to apparent increases of ECC15 *pyrE* gut persistence. Given the roles of uracil and its derivative in a wide array of enzymatic events as coenzyme and regulator [39–41] as well as in RNA synthesis as a building block, we suspect that the lack of uracil biosynthesis in the bacteria might cause physiological bottlenecks, hindering normal growth in the gut. In parallel with this view, supplementation of ECC15 *pyrE* with uracil raised CFU in the *TrpA1*^*ins*^ not *wcs* guts albeit at a delayed time point, 8 hrs (Fig 5D, *right*). Uracil supplementation might aid ECC15 *pyrE* to grow faster in the gut, but at the same time trigger the *Duox* pathway to drive the *TrpA1*-dependent expulsion. Because residing time or growth of ECC15 in the gut can be greatly influenced by the commensal microbiome that can differ in the genotypes of interest, germ-free animals were generated and tested for defecation induction by uracil (S11 Fig) and gut persistence (Fig 5E). These experiments produced results very similar to those with non-germ-free animals, suggesting that the *TrpA1-* and uracil-dependent defecation is little affected by pre-established microbiome. To examine the extent to which *TrpA1-*dependent defecation is critical for elimination of ECC15 from the animals, ECC15 that stayed in either gut of *wcs* or *TrpA1*^*ins*^ for 5 hrs were assessed for their HOCl resistance by incubation of the bacteria with varying concentrations of HOCl for 30 min. The resistance was similar between the bacteria isolated from *wcs* and *TrpA1*^*ins*^ guts (S12A Fig). However, in three out of eight experiments, ECC15 from *TrpA1*-deficient guts showed colonies that appeared 2 days after spotting, while ECC15 from *wcs* guts did not in all eight experiments (S12B Fig). This acquired resistance to HOCl was transient, as the surviving colonies did not grow again with the second incubation in 10 ppm of HOCl. Although the results did not reach statistical significance (Fisher’s exact test, p = 0.2), the opportunistic survival of ECC15 from *TrpA1*^*ins*^ guts at the HOCl concentration that prevented the growth of ECC15 from *wcs* guts suggests that the timely clearance of the pathogenic bacteria might be critical to eliminate the opportunity to gain the ability to survive the Duox defense system in the gut.

### The deficiency of *TrpA1* exacerbates mortality of the *rel*^*e20*^ mutant orally ingesting ECC15

To examine the impact of uracil-evoked defecation in the mortality rate of flies, flies were subjected to ingestion of ECC15 at OD600 of 10 in 500 mM sucrose, and monitored for survival (Fig 5F). Although *TrpA1*^*ins*^ showed resistance to ECC15 similar to *wcs*, the mortality rate of *rel*^*e20*^ was significantly higher than that of *wcs*. Interestingly, the double mutant flies lacking both *rel* and *TrpA1* (*TrpA1*^*ins*^, *rel*^*e20*^) were least resistant to orally ingested ECC15, revealing that uracil-induced defecation is important for the survival of immune-compromised animals. This enhanced mortality in *TrpA1*^*ins*^, *rel*^*e20*^ was not observed when ECC15 *pyrE* was ingested, suggesting the role of uracil excreted from ECC15 in resisting to the bacteria (Fig 5G). *TrpA1*^*ins*^ showed extended life span with the ECC15 *pyrE* ingestion beyond that of *wcs*. The extended life span of *TrpA1*^*ins*^ ingesting ECC15 *pyrE* appears to stem from genetic factors other than *TrpA1*, as it was not rescued by reintroduction of the *TrpA1* gene (S12C Fig).

In previous studies, it has been shown that bacterially released uracil stimulates production of microbicidal reactive chlorine by Duox, thus killing bacterial pathogens in the gut. Microbes are, however, often equipped with dedicated chlorine-responsive machineries mounting reactive chlorine resistance [42–44]. Therefore, intestinal bacteria steadily subjected to reactive chlorine might develop resistance to the toxicity, which could be a detrimental outcome for host health. In this regard, expelling pathogenic bacteria from the gut would be a preemptive measure curtailing the possibility. Our results suggest that the *TrpA1*-mediated response to reactive chlorine helps flies restrain bacterial pathogens from acquiring reactive chlorine resistance by promoting pathogen expulsion. For such processes, *TrpA1* expression is required in enteroendocrine cells, where TRPA1 activation might promote release from the EECs of intestinal hormones or transmitters that have yet to be identified. It has been reported in mammals that TRPA1 in enterochromaffin cells is responsible for serotonin release by oxidative stress in the gut [13] and that serotonin biosynthesis is regulated by commensal bacteria thus affecting intestinal motility and hemostasis [14]. It would be interesting to examine in the future if the role of serotonin or equivalent signaling messengers from EECs are conserved for uracil-evoked defecation in *Drosophila*, and have physiological implications in regards of host/microbiome interaction.

Reactive chlorine species are not only highly bactericidal but also harmful to host cells. Although the gut contains a host-protecting antioxidant system [45], sustained Duox activation by uracil-releasing opportunistic pathobionts causes chronic inflammation through gut cell apoptosis [8]. In order to restrict such hazardous effects of reactive chlorine, *Drosophila* guts may control the uracil-elicited HOCl production at which level opportunistic pathogens are eliminated but cytotoxicity is insignificant. Our results reveal that low amounts of unstable reactive chlorine can be readily received by TRPA1(A)10b. Thus, the exceptional activation kinetics and enhanced sensitivity to HOCl of the TRPA1(A)10b isoform reduce the risk of inflammation which may otherwise result from the need of prolonged exposure to excessive reactive chlorine. Our comparative analysis of human TRPA1 with *Drosophila* TRPA1 isoforms showed that human TRPA1 is inferior to TRPA1(A)10b in terms of NaOCl response kinetics and sensitivity (S4 Fig), suggesting that tuning of mammalian TRPA1 for a sufficient HOCl responsiveness may be required for the Duox-dependent defecation in mammalian intestines. Indeed, a newly identified RNA splice variant encodes a TRPA1 isoform that heightens the sensitivity to reactive chemicals by forming heterotetramers with the classical TRPA1 in mice [46]. It would be intriguing to examine that the heterotetramers of mouse TRPA1 show similar functional shifts in response to NaOCl, while the alternative domain occurs in a region spatially distinct that of *Drosophila* TRPA1. A recent study proposed that TRPA1(A)10b is a potential receptor for H_2_O_2_ [47] which is the intermediate Duox product, while its H_2_O_2_ responsiveness appeared to be similar to that of TRPA1(A)10a. Considering that *TrpA1(A)10b* but not *TrpA1(A)10a* is required for Duox-dependent defecation in EECs, HOCl rather than H_2_O_2_ is the key player resulting from uracil-induced Duox upregulation.

How bacteria affect host defecation in mammals is not entirely understood. Mammals express Duox in mucosal epithelia, where bacteria densely colonize [6]. At the same time, mammalian TRPA1s function in neurons and enterochromaffin cells closely associated with the mucosal tissues [13,18], implying potential crosstalk between Duox and TRPA1 in mammals. Irritable bowel syndrome (IBS) is a disease characterized by dysregulated defecation, which can be caused by an imbalance between gut microbiota and host immunity [48]. Moreover, visceral pain and dysregulated defecation are two major symptoms of IBS, which are reminiscent of *Drosophila* TRPA1 functions in chemical nociception [19,22] and defecation (described here). Our discovery of a chemical link between Duox and TRPA1 in flies raises the possibility that oxidative stress from bacterially stimulated mucosal Duox may interact with TRPA1 in mammals to trigger defense responses such as increased bowel movement, the dysregulation of which may lead to pathological states such as IBS.

## Materials and Methods

### Fly strains

Site-specific transgenesis [49] was used to introduce the *UAS-TrpA1(A)10b* transgene into the genome at the same site as *UAS-TrpA1(A)10a* [22] and *UAS-TrpA1(B)* [19], attp16 [50] in order to control transgene position effects. Other fly lines were mostly acquired from the Bloomington *Drosophila* Stock Center, IN, USA. The *da-Gal4* line is a kind gift from Won-Jae Lee at Seoul National University, *TrpA1(A)-Gal4* [25] from Daniel Tracey at Duke University and *TrpA1*^*1*^ and *TrpA1*^*Gal4*^ [29] from Craig Montell at Johns Hopkins University. The *UAS-Duox*^*RNAi*^ and *UAS-TrpA1*^*RNAi*^ used in this study were the fly stocks of #33975 and #31384, respectively, from the Bloomington *Drosophila* Research Stock Center. These RNAi transgenic lines were generated by the Transgenic RNAi Resource Project (TRiP) [51]. Axenic animals were generated for defecation analyses and gut persistence experiments from bleached embryos, which were subsequently transferred to autoclaved media in fly vials. The first and second generations of the germ-free flies were used for experiments in the study.

### Defecation assay

In order to measure defecation frequencies, 12 to 15 male flies were used 4 to 6 days after eclosion. Female defecation is affected by reproduction status [31], which may mask the effect of uracil on defecation. Siblings were divided into two groups and starved for 16 hrs in humidified conditions at room temperature. One group of flies was fed with 500 mM sucrose solution, and the other was with either uracil (U1128, Sigma Aldrich, MO, USA) or N-methylmaleimide (NMM; 389412, Sigma Aldrich, MO, USA) in addition to 500 mM sucrose. Uracil concentrations used in the dose dependence experiment were 0.2, 20, 20,000 and 100,000 nM (Fig 1*B*). For the rest of uracil-induced defecation experiments, 20,000 nM was used. For the convenience of counting defecation spots left dried on the inner wall of regular fly vials (AS-514, Fisher Scientific, MA, USA), 0.01% food colorant Brilliant Blue FCF (Blue 1, 861146, Sigma Aldrich, MO, USA) was added to all ingested solutions. Flies were fed in a modified capillary feeder (Café) configuration [52], in which sucrose solutions in five-microliter graded capillary tubes (2920107 Marienfeld, Lauda-Königshofen, Germany) were offered for 2 hrs in a fly vial. Subsequently, the flies were removed and further flipped to a new vial every hour for 8 hrs in total including the 2 hr ingestion (Fig 1A). In this way, we could assess the amount of food ingested and normalize the defecation frequency with respect to the level of ingestion. Humidified conditions between 50 and 70% were maintained throughout ingestion and defecation. The ingested amount was expressed in length (millimeter) of the change in food height in the capillary tube (15 mm/ μl).

To assess the defecation responses to Ecc15 strains, each strain was precultured for 8 hrs at 30°C in 4 ml of LB broth with the appropriate antibiotics. The preculture was transferred to a larger volume of LB at a ratio of 1:100 for the overnight main culture. The harvested bacteria were washed once in PBS (10 mM Na_2_HPO_4_, 1.8 mM KH_2_PO_4_, 140 mM NaCl, 2.7 mM KCl, pH 7.3) and resuspended in 500 mM sucrose solution to the optical density (OD) of 10 at 600 nm to be offered via the capillaries to flies.

To estimate the total amount of defecation, the flies were offered for two hours with conditioned sucrose solutions containing 0.5% brilliant blue FCF and allowed to defecate in vials for 6 hours. The inside of each vial was washed with 2.5 ml of PBS. The absorbance at 628 nm was determined by spectrometry for comparative quantitation of the dye. The absorbance was then divided by the ingested amount assessed from migration of sucrose solution meniscus in the feeding capillary for normalization.

### Evaluation of mortality rates for flies that ingested ECC15 strains

Twenty male flies were transferred every day to a new vial containing a round 3M paper soaked in 500 mM sucrose solution with ECC15 at OD10. The bacteria culture was prepared freshly every day for consistency of the survival experiments. The inactive flies were counted before the transfer. The data were plotted and analyzed by means of the Kaplan Meier analysis offered in Sigmaplot12.0.

### *In vivo* bacterial persistence assay

To evaluate bacterial persistence in the gut, spectinomycin-resistant Ecc15-GFP cells suspended at OD 10 in 500 mM sucrose solution were orally fed to the flies in the Café configuration. The gut was dissected out at the time points indicated in Fig 4, and ground in PBS followed by vigorous vortexing for 1 min. The released gut bacteria was serially diluted and plated on spectinomycin LB-agar dishes. Mean CFUs averaged from three serial dilutions were used for data analysis.

### Immunohistochemistry and confocal imaging

The previously published gut immunocytochemistry protocol was used [16] with minor modifications. Briefly, whole abdomens were immunostained before the intestines were dissected out and mounted for imaging. Whole abdomens were separated and punctured for reagent infiltration to the intestine in phosphate buffered saline with 0.2% Triton X-100 (PBS-T, pH 7.2), and fixed in 4% paraformaldehyde in PBS-T overnight at 4°C. After 3 washes with PBS-T, samples were blocked with 3% goat serum in PBS-T for at least 30 min. Abdomens were incubated with primary antibodies or antisera for 1–2 days at 4°C, washed with PBS-T, and incubated with secondary antibodies overnight at 4°C. The primary antibodies used were rat anti-TRPA1 (1:200) [19,22,37], rabbit anti-GFP (1:1000, Life Technologies, CA, USA), anti-Prospero (1:10, Developmental Studies Hybridoma Bank at the University of Iowa). The secondary antibodies used were Alexa Fluor Cy3-labeled goat anti-rat (1:1000, Jackson Laboratory, ME, USA), Alexa Fluor 488-labeled goat anti-rabbit (1:200, Life Technologies, CA, USA), and Alexa Fluor 568-labeled goat anti-mouse IgG (1:1000, Life Technologies, CA, USA). A Zeiss LSM 700 laser-scanning confocal microscope was used to acquire images of immunostained samples.

### Functional characterization of TRPA1 in *Xenopus* oocytes

TRPA1 currents in *Xenopus laevis* oocytes evoked by application of chemicals were recorded by two-electrode voltage clamping as previously described [19,22]. Briefly, surgically prepared ovaries were subjected to collagenase treatment to free the cells from the tissue. One day after injection of 50 nl of *TrpA1* cRNA, oocytes were perfused in the recording solution (96 NaCl, 1 KCl, 1 MgCl_2_, 5 HEPES, pH 7.6 in mM). NaOCl (425044, Sigma Aldrich, MO, USA) was freshly diluted immediately before experiments. TRPA1-expressing oocytes exhibited resting membrane potentials between -10 and -50 mV. Voltage was initially held at -60 mV, and a 300-ms voltage ramp from -60 to +60 mV was applied every second by the GeneClamp 500B amplifier (Molecular Devices, CA, USA) during data acquisition (Digidata 1440A, Molecular Devices, A, USA). To determine NaOCl sensitivities of the TRPA1 isoforms, currents were recorded till reaching steady state after evoked by a concentration of NaOCl, rather than fixing the application time across the experiments. Data points from dose dependence experiments were normalized with the respect to the current amplitude achieved by 100 ppm NaOCl, and fitted to the Hill equation by Sigmaplot12. Since current traces were often unable to be fitted to the single exponential equation, Time to the 70% maximum current at each NaOCl concentration was determined.

### NaOCl resistance assay

Five hours after start of ECC15-GFP at OD 10 in 500 mM sucrose, five guts per genotype were dissected out and ground in 0.2 ml of PBS. Each aliquot of 0.04 ml of ground guts was incubated with either 0, 0.1, 1, 5 or 10 ppm of NaOCl for 30 min at room temperature in 1 ml of PBS. Subsequently, the surviving bacteria were spot-titrated on spectinomycin LB agar plates with serial dilution in PBS.

### *In vivo* recording of gustatory neurons

Extracellular recordings of gustatory neurons in L-bristles were conducted as previously described in detail [22].

### Statistics

Student’s t-test and ANOVA Tukey multiple comparison and ANOVA repeated measures tests were performed with Sigmaplot12.

### Molecular biology

Complementary DNA was prepared from dissected fly guts using the RETROscript kit (AM1710, Life Technologies, CA, USA). Primers used to determine exons encoding the N-terminus were described previously [22]. Regarding exon 10 splicing, the following primers were used for RT-PCR (Fig 2*C*).

E10com-F: 5’-GTGGACAAGGATGGGAAC-3’

10a-R: 5’-CTCTCCGGTTTTCTCATCA-3’

10b-R: 5’-GGTAGGGCCAAAACGAA-3’

### Quantitation of HOCl with R19S

The response profile of R19S to the concentration range of NaOCl used in the study was determined with Deltascan (PTI, USA). For confocal scanning of the R19S fluorescence signal (LSM710, Zeiss, Germany), sucrose solutions containing 10 μM R19S with or without 20 nM uracil or with uracil and DTT or with NMM were fed to flies in Café configuration for 1 hr. Subsequently, the intestines of the flies were dissected out and fixed in 2% paraformaldehyde for 15 min. The mean pixel intensity was measured by Zen Pro (Zeiss, Germany) in a middle part of the anterior midgut captured with Plan-Apochromat 20x/0.8 M27 (Zeiss, Germany).

## Supporting Information

S1 Fig *TrpA1* necessary for uracil-induced defecation is unrelated to the *imd* pathway and food ingestion. (**A-E)** Defecation spot numbers normalized to ingestion amounts (indicated as output/input, spots/mm) are presented in addition to fold change of defecation results. The ingestion amount was assessed as the length change of food filling 5 microliter glass capillary tubes (15 mm/microliter). n = 6–14 **(A-E**). (**D**) *Duox* RNAi knockdown by pan-neuronal *appl-Gal4* did not abolish uracil-dependent defecation (n = 7–11). *Left*: The results presented as “Output/Input”, *Right*: fold change of defection derived from the *Left* panel (n = 6–14). (**F**) The ingestion amount per animal was analyzed across genotypes (n = 6–18). Ingestion with uracil or DTT did not significantly alter the ingestion amounts. Only statistically significant comparisons were marked: *, p<0.05, **, p<0.01, ***, p<0.001. Student’s *t-*test or Tukey.
(PDF)

S2 Fig Spectral quantitation of uracil-dependent defecation. (A) Fecal spot sizes are not significantly changed by uracil ingestion. Sucrose solution with (+uracil) or without uracil (-uracil) was fed to indicated genotypes which were at the same time allowed to defecate on the inside wall of a cuvette for providing flat surface. The fecal spots were scanned and the area sizes were assessed by Image J. (**B**) Indicated fly alleles were fed with 500 mM sucrose solution with 0.5% brilliant blue FCF containing indicated ECC15 strains, and allowed to defecate in fly vials for 8 hrs in total. Fecal spots were washed with 2.5 ml PBS. (**C**) Fecal spectral absorbances at 628 nm normalized with respect to input. Uracil (upper) or ECC15 WT ingestion (lower) resulted in increased spectral absorbance, compared to sucrose only or ECC15 *pyrE* ingestion, respectively. *: p<0.05 and ***:p<0.001, Student *t*-test. The number of experiments is indicated at the base of the graphs in grey.
(PDF)

S3 Fig Uracil does not directly activate TRPA1 and is not chemically inactivated by incubation with DTT. (**A** and **B**) Prolonged application (6 min) of 0.02 mM uracil did not evoke TRPA1 currents in *Xenopus* oocytes expressing either TRPA1(A)10a or TRPA1(A)10b, while the well known TRPA1 activator, NMM, did. The currents were recorded with 300-ms voltage ramps between -60 and +60 mV every one second. (**C**) Summary of uracil responses appraised at +60 mV in the oocytes (n = 4–5). (**D**) Gustatory choice test results (n = 4–7). *TrpA1* in the taste neurons mediates avoidance behavior to TRPA1 agonists [19]. Twenty micromolar uracil in the sucrose solution insignificantly shifted feeding towards the sucrose-only condition. The capillary feeder (Café) assay was used. One hundred millimolar sucrose solutions either with or without indicated chemicals at the given concentrations were offered through glass capillary tubes at the same time. The decreased volume in sucrose tubes minus that of tubes with sucrose+indicated chemical was divided by the volume of total consumption to score the “Avoidance Index”. ***, p<0.001. Tukey. (**E-G**) Uracil at 0.1 mM was mixed with 10 mM DTT, and incubated for 2 hrs at room temperature, after which the spectral absorbance was determined. Single chemical conditions were also subjected to the test as controls. (**E**) Spectral absorbance of indicated chemicals. Different colors indicate independent experiments (n = 3). Arrows indicate the absorbance peak of uracil at 260 nm. Inset (Top and Bottom panels): magnification for uracil peak absorbance. (**F**) The peak uracil absorbance at 260 nm of three different conditions. (**G**) The peak DTT absorbance at 205 nm.
(PDF)

S4 Fig The HOCl production in the gut was monitored with the use of the HOCl-specific dye R19S. (**A**) Spectral evaluation of R19S in the range of NaOCl concentrations from 0.1 to 100 ppm. *Left*, A representative NaOCl dose-dependent change of R19S fluorescence. *Right*, The averaged graph of R19S fluorescence at indicated NaOCl concentrations (n = 4). (**B**) Typical confocal images of intestines with R19S. Experimental conditions were indicated at the left side of images. A part of the anterior midgut indicated as a red box in (**C**) is shown for each experiment. Field of view: 420 microns. (**C**) An R19S image of the midgut and hindgut from a fly that ingested uracil. R19S fluorescence was observed throughout the intestine, suggesting that HOCl production is not spatially limited. The red box indicates approximate location of the part shown in (**B**). (**D**) Ingestion of the R19S and 0.1 ppm NaOCl premixture yields the intensity and pattern of R19S fluorescence similar to the guts from animals that ingested 20 nM uracil. *Right*, A composite image illustrating the fluorescence distribution in the mid- and hindgut. (**E**) Averaged data are presented as bar graphs. Letters indicate significantly distinct groups. ANOVA Tukey, p<0.001. The number of experiments is indicated at the base of the graphs in grey.
(PDF)

S5 Fig Deficits in uracil-dependent defecation by genetic manipulations via the *Gal4/UAS* system are not due to developmental impairments. (**A**) Schematic diagram of the temporal control of *Gal4/Gal80*^*ts*^-dependent transcription. (**B**-**D**) Genetic interventions such as *Duox* (B) or *TrpA1* (D) RNAi knockdown and *Jafrac1* overexpression (C) by indicated *Gal4* lines. Non-permissive temperature at 30°C disrupts transcriptional suppression of GAL80^ts^ and allows GAL4 to drive transcription, while GAL80^ts^ restricts transcription at permissive temperatures such as 18°C. **: p<0.01, ANOVA Tukey.
(PDF)

S6 Fig Higher magnification images of Fig 2A. (PDF)

S7 Fig TRPA1 is expressed in enteroendocrine cells important for uracil-dependent defecation. (**A**) Schematic illustration of the gut regions stained with anti-TRPA1 antibody. (**B**) *TrpA1(A)-Gal4* cells are enteroendocrine cells marked with anti-Prospero, except for large cells in the middle midgut that appear to be enterocytes (not shown). (**C**) *TrpA1* RNAi knockdown in *TrpA1(A)-Gal4* cells presented as “Output/Input” (n = 5–8).
(PDF)

S8 Fig Anti-TRPA1 staining reveals TRPA1-positive EECs not associated with intestinal stem cell (A) and enteroblast markers (B) and the *TrpA1* RNAi knockdown effect (C). TRPA1 staining results for RNAi knockdowned animals are summarized in the table (**C**).
(PDF)

S9 Fig NaOCl response parameters of *Drosophila* TRPA1 isoforms and human TRPA1 heterologously expressed in oocytes, and differential HOCl responses of TRPA1 isoforms ectopically expressed in sugar-sensing *Gr5a* cells. (**A**-**B**) Dose dependence (*Left)* and current voltage relationship (*Right*) of the temperature-sensitive *Drosophila* TRPA1 isoform, TRPA1(B)10a (**A**), and human TRPA1 (hsTRPA1, panel **B**). (**C**) Summarized dose dependence of hsTRPA1 (n = 4–10) and TRPA1(B)10a (n = 4–6). hsTRPA1 has a dose dependence profile very similar to TRPA1(B)10a. (**D**) Time constants from dose dependence experiments reveal that hsTRPA1 exhibits faster activation than TRPA1(B)10a but slower activation thanTRPA1(A) at 10 ppm NaOCl (n = 4–10). (**E**) Time constants of *Drosophila* TRPA1 isoforms and human TRPA1 (hsTRPA1) at -60 (*Upper*) and +60 mV (*Lower*) (n = 4–9). Shaded boxes indicate the concentrations that failed to generate sufficient currents for time constant determination. Note that only TRPA1(A)10b had time constants measured for 0.1 ppm. (**F**) EC50s from various TRPA1s at -60 and +60 mV (n = 4–10). EC50 of hsTRPA1 is comparable to that measured by a previous report [18]. *: p<0.05, ***: p<0.001, Tukey test vs. hsTRPA1 (**D**). ND: not determined. (**G**) Current amplitudes evoked by 100 ppm HOCl for estimation of expression in oocytes (n = 4–13). (**H**) L-bristles of animals with the indicated genotypes were examined for their NaOCl responsiveness. Note that *Gr5a>TrpA1(A)10a* taste neurons hardly showed action potentials to NaOCl 100 ppm, while responding to the immediately following NMM contact. In contrast, *Gr5a* cells expressing TRPA1(A)10b showed robust spikes from both sweet and water cells. The noisy low level spikes frequently appeared when the sensilla contact with NaOCl, probably due to its reactivity. (**I**) Summary of 30 sec-averaged spike frequencies evoked by NaOCl in L-bristles of flies used in (**H**) (n = 4–8). ***: p≤0.001, Tukey test.
(PDF)

S10 Fig TRPA1(A)10b responds fast and sensitively to citronellal compared to TRPA1(A)10a, when expressed in frog oocytes. The non-covalent agonist citronellal opens TRPA1(A)10b faster and more sensitively than TRPA1(A)10a. Typical current recording with increasing concentrations of citronellal for TRPA1(A)10a (**A**) and TRPA1(A)10b (**B**). (**C**) Citronellal dose dependences of the two isoforms. (D) Time to 70% amplitude at each concentration was plotted as function of citronellal concentrations.
(PDF)

S11 Fig ECC15-dependent defecation in germ-free *Drosophila melanogaster*. *Upper*, Output/input results were presented for indicated genotypes. Defecation frequencies were normalized to the feeding amounts after ingestion of either ECC15 WT or *pyrE*. *Lower*, The results in the *Upper* panel were displayed as fold change of defecation for estimation of fold increase of defecation by uracil from ECC15. **: p<0.01, ANOVA Tukey or Student *t-*test.
(PDF)

S12 Fig Transiently acquired NaOCl resistance of ECC15 from the *TrpA1*^*ins*^ gut and mortality rates of flies upon ingestion of ECC15 *pyrE*. (**A**) NaOCl resistance was assessed with ECC15-GFP that previously resided in *wcs* or *TrpA1*^*ins*^ guts in the range of indicated concentrations and is not significantly different between the two genotypes. (**B**) ECC15-GFP from *TrpA1*^*ins*^ guts opportunistically survive the NaOCl concentration that eliminated the growth of the bacteria from *wcs* guts. (**C**) Enhanced survival to ingested ECC15 *pyrE* of *TrpA1*^*ins*^ is not rescued by reintroduction of the *TrpA1* genomic DNA, suggesting that the resistance *TrpA1*^*ins*^ exhibited might have originated from its genetic background.
(PDF)
