# Supplementary material for: TrpA1 Regulates Defecation of Food-Borne Pathogens under the Control of the Duox Pathway
Source: PLoS Genet. 2016 Jan 4;12(1):e1005773. doi: 10.1371/journal.pgen.1005773 (PMC4699737; doi:10.1371/journal.pgen.1005773)
Supplement: S1 Fig — (A-E) Defecation spot numbers normalized to ingestion amounts (indicated as output/input, spots/mm) are presented in addition to fold change of defecation results. The ingestion amount was assessed as the length change of food filling 5 microliter glass capillary tubes (15 mm/microliter). n = 6–14 (A-E). (D) Duox RNAi knockdown by pan-neuronal appl-Gal4 did not abolish uracil-dependent defecation (n = 7–11). Left: The results presented as “Output/Input”, Right: fold change of defection derived from the Left panel (n = 6–14). (F) The ingestion amount per animal was analyzed across genotypes (n = 6–18). Ingestion with uracil or DTT did not significantly alter the ingestion amounts. Only statistically significant comparisons were marked: *, p<0.05, **, p<0.01, ***, p<0.001. Student’s t-test or Tukey. (PDF) [file pgen.1005773.s001.pdf]

Figure S1.

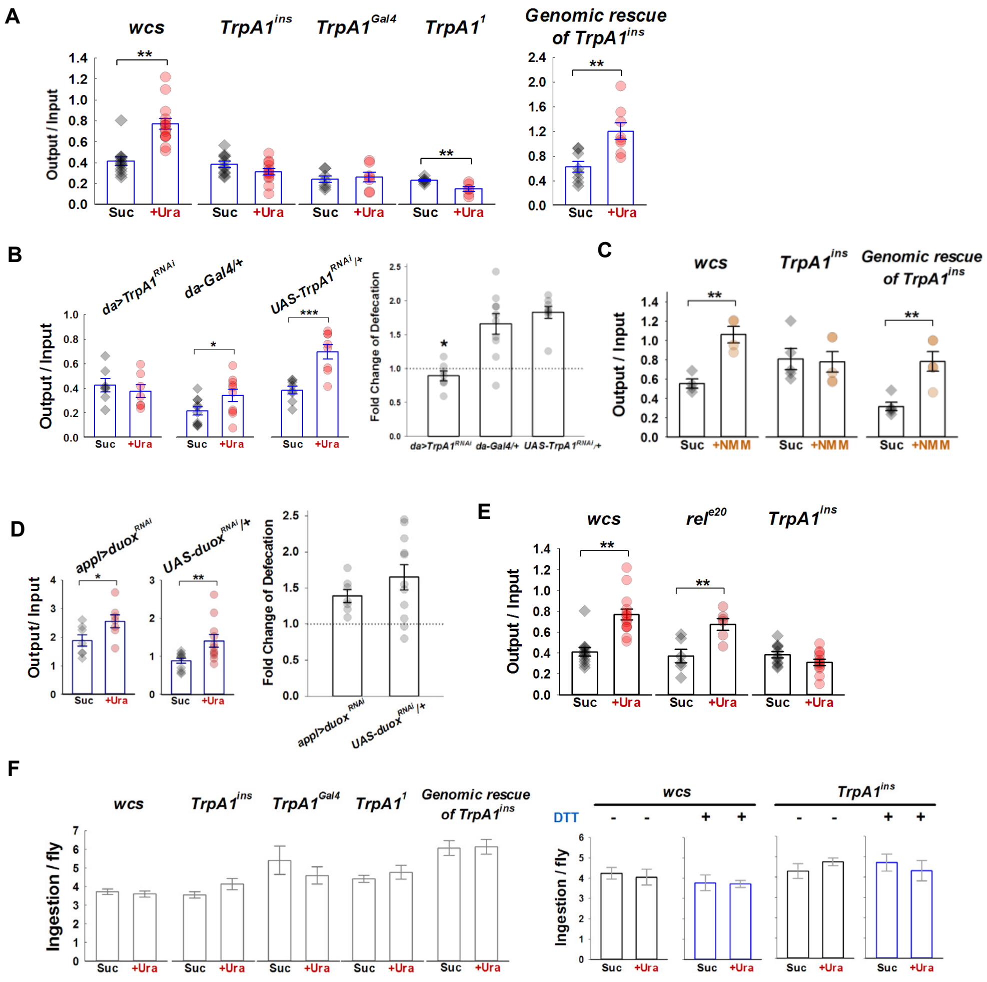

Figure S1. ***TrpA1*** **necessary for uracil-induced defecation is unrelated to the** ***imd*** **pathway and food ingestion.** **(A-E)** Defecation spot numbers normalized to ingestion amounts (indicated as output/input, spots/mm) are presented in addition to fold change of defecation results. The ingestion amount was assessed as the length change of food filling 5 microliter glass capillary tubes (15 mm/microliter). n=6-14 **(A-E).** (**D**) *Duox* RNAi knockdown by pan-neuronal *appl-Gal4* did not abolish uracil-dependent defecation (n=7-11). *Left:* The results presented as “Output/Input”, *Right:* fold change of defection derived from the *Left* panel (n=6-14). (**F**) The ingestion amount per animal was analyzed across genotypes (n=6-18). Ingestion with uracil or DTT did not significantly alter the ingestion amounts. Only statistically significant comparisons were marked: *, p<0.05, **, p<0.01, ***, p<0.001. Student’s *t*-test or Tukey.
