# Supplementary material for: TrpA1 Regulates Defecation of Food-Borne Pathogens under the Control of the Duox Pathway
Source: PLoS Genet. 2016 Jan 4;12(1):e1005773. doi: 10.1371/journal.pgen.1005773 (PMC4699737; doi:10.1371/journal.pgen.1005773)
Supplement: S2 Fig — (A) Fecal spot sizes are not significantly changed by uracil ingestion. Sucrose solution with (+uracil) or without uracil (-uracil) was fed to indicated genotypes which were at the same time allowed to defecate on the inside wall of a cuvette for providing flat surface. The fecal spots were scanned and the area sizes were assessed by Image J. (B) Indicated fly alleles were fed with 500 mM sucrose solution with 0.5% brilliant blue FCF containing indicated ECC15 strains, and allowed to defecate in fly vials for 8 hrs in total. Fecal spots were washed with 2.5 ml PBS. (C) Fecal spectral absorbances at 628 nm normalized with respect to input. Uracil (upper) or ECC15 WT ingestion (lower) resulted in increased spectral absorbance, compared to sucrose only or ECC15 pyrE ingestion, respectively. *: p<0.05 and ***:p<0.001, Student t-test. The number of experiments is indicated at the base of the graphs in grey. (PDF) [file pgen.1005773.s002.pdf]

Figure S2

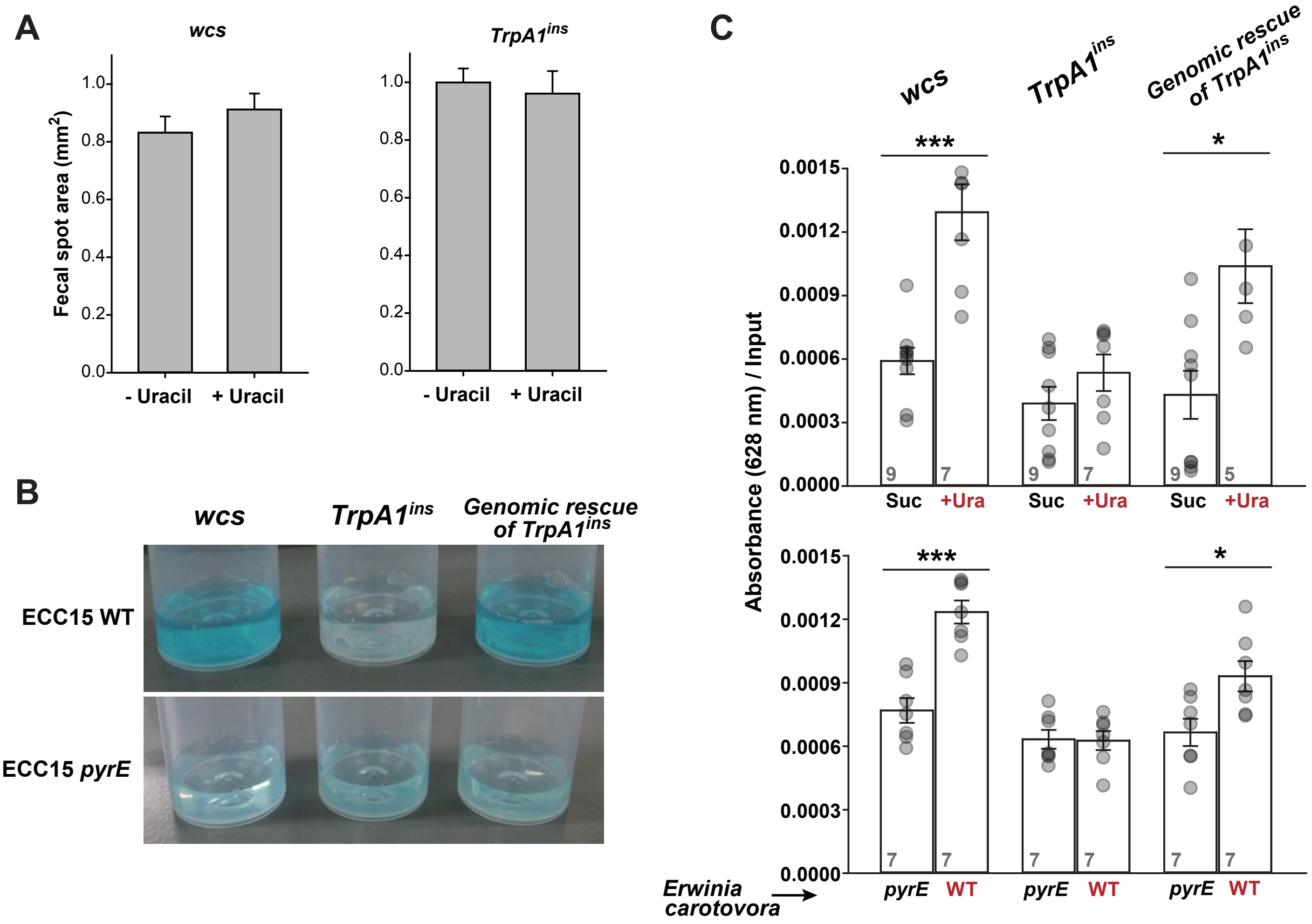

Figure S2. Spectral quantitation of uracil-dependent defecation. (A) Fecal spot sizes are not significantly changed by uracil ingestion. Sucrose solution with (+uracil) or without uracil (-uracil) was fed to indicated genotypes which were at the same time allowed to defecate on the inside wall of a cuvette for providing flat surface. The fecal spots were scanned and the area sizes were assessed by Image J. (**B**) Indicated fly alleles were fed with 500 mM sucrose solution with 0.5% brilliant blue FCF containing indicated ECC15 strains, and allowed to defecate in fly vials for 8 hrs in total. Fecal spots were washed with 2.5 ml PBS. (**C**) Fecal spectral absorbances at 628 nm normalized with respect to input. Uracil (upper) or ECC15 WT ingestion (lower) resulted in increased spectral absorbance, compared to sucrose only or ECC15 *pyrE* ingestion, respectively. *: $p<0.05$ and ***:$p<0.001$, Student *t*-test. The number of experiments is indicated at the base of the graphs in grey.
