# Supplementary material for: TrpA1 Regulates Defecation of Food-Borne Pathogens under the Control of the Duox Pathway
Source: PLoS Genet. 2016 Jan 4;12(1):e1005773. doi: 10.1371/journal.pgen.1005773 (PMC4699737; doi:10.1371/journal.pgen.1005773)
Supplement: S3 Fig — (A and B) Prolonged application (6 min) of 0.02 mM uracil did not evoke TRPA1 currents in Xenopus oocytes expressing either TRPA1(A)10a or TRPA1(A)10b, while the well known TRPA1 activator, NMM, did. The currents were recorded with 300-ms voltage ramps between -60 and +60 mV every one second. (C) Summary of uracil responses appraised at +60 mV in the oocytes (n = 4–5). (D) Gustatory choice test results (n = 4–7). TrpA1 in the taste neurons mediates avoidance behavior to TRPA1 agonists [19]. Twenty micromolar uracil in the sucrose solution insignificantly shifted feeding towards the sucrose-only condition. The capillary feeder (Café) assay was used. One hundred millimolar sucrose solutions either with or without indicated chemicals at the given concentrations were offered through glass capillary tubes at the same time. The decreased volume in sucrose tubes minus that of tubes with sucrose+indicated chemical was divided by the volume of total consumption to score the “Avoidance Index”. ***, p<0.001. Tukey. (E-G) Uracil at 0.1 mM was mixed with 10 mM DTT, and incubated for 2 hrs at room temperature, after which the spectral absorbance was determined. Single chemical conditions were also subjected to the test as controls. (E) Spectral absorbance of indicated chemicals. Different colors indicate independent experiments (n = 3). Arrows indicate the absorbance peak of uracil at 260 nm. Inset (Top and Bottom panels): magnification for uracil peak absorbance. (F) The peak uracil absorbance at 260 nm of three different conditions. (G) The peak DTT absorbance at 205 nm. (PDF) [file pgen.1005773.s003.pdf]

Figure S3

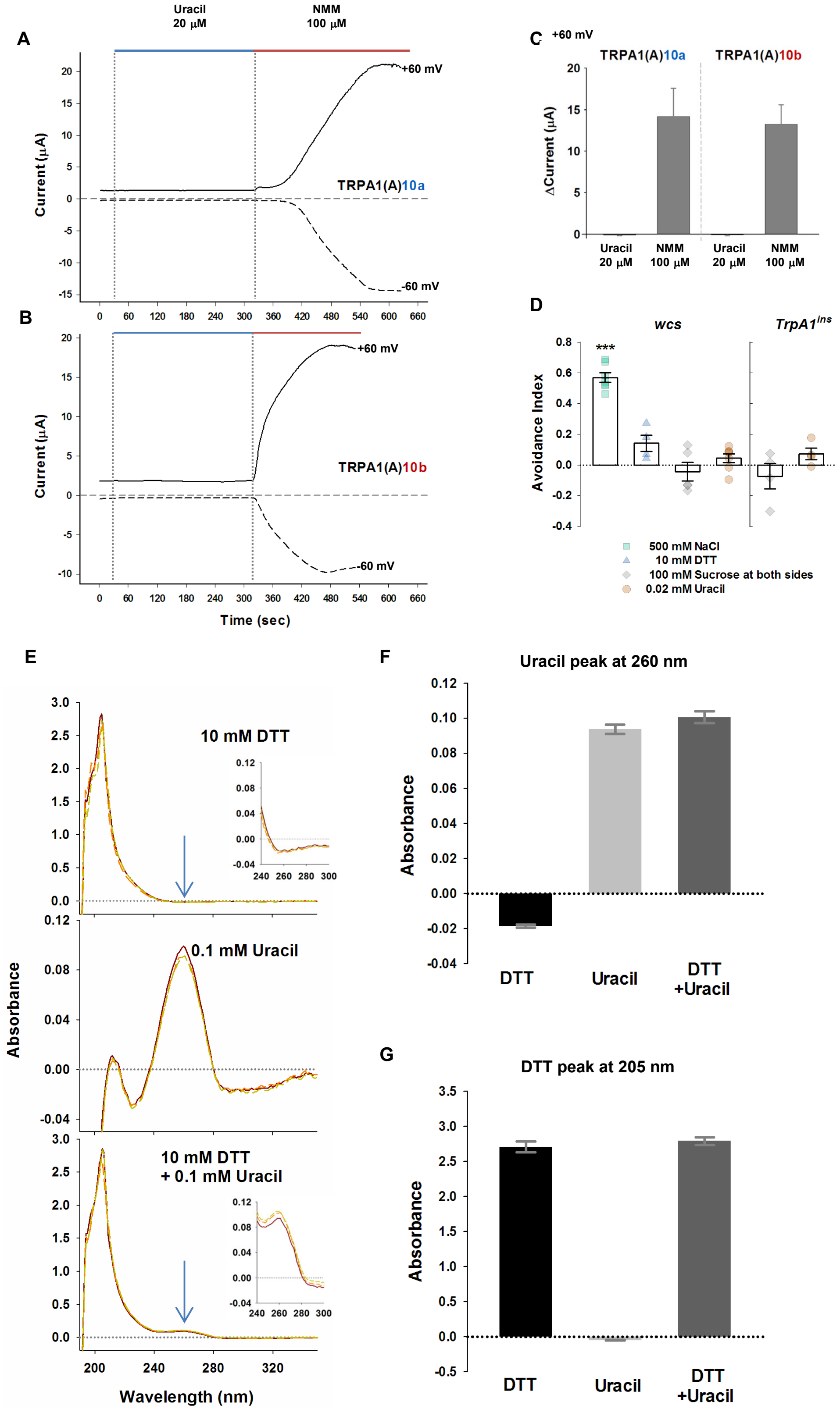

Figure S3. Please check the next page for full legend.

Figure S3. Uracil does not directly activate TRPA1 and is not chemically inactivated by incubation with DTT. (**A** and **B**) Prolonged application (6 min) of 0.02 mM uracil did not evoke TRPA1 currents in *Xenopus* oocytes expressing either TRPA1(A)10a or TRPA1(A)10b, while the well known TRPA1 activator, NMM, did. The currents were recorded with 300-ms voltage ramps between -60 and +60 mV every one second. (**C**) Summary of uracil responses appraised at +60 mV in the oocytes (n=4-5). (**D**) Gustatory choice test results (n=4-7). *TrpA1* in the taste neurons mediates avoidance behavior to TRPA1 agonists [19]. Twenty micromolar uracil in the sucrose solution insignificantly shifted feeding towards the sucrose-only condition. The capillary feeder (Café) assay was used. One hundred millimolar sucrose solutions either with or without indicated chemicals at the given concentrations were offered through glass capillary tubes at the same time. The decreased volume in sucrose tubes minus that of tubes with sucrose+indicated chemical was divided by the volume of total consumption to score the "Avoidance Index". ***, $p<0.001$. Tukey. (**E-G**) Uracil at 0.1 mM was mixed with 10 mM DTT, and incubated for 2 hrs at room temperature, after which the spectral absorbance was determined. Single chemical conditions were also subjected to the test as controls. (**E**) Spectral absorbance of indicated chemicals. Different colors indicate independent experiments (n=3). Arrows indicate the absorbance peak of uracil at 260 nm. Inset (Top and Bottom panels): magnification for uracil peak absorbance. (**F**) The peak uracil absorbance at 260 nm of three different conditions. (**G**) The peak DTT absorbance at 205 nm.
