# Supplementary material for: TrpA1 Regulates Defecation of Food-Borne Pathogens under the Control of the Duox Pathway
Source: PLoS Genet. 2016 Jan 4;12(1):e1005773. doi: 10.1371/journal.pgen.1005773 (PMC4699737; doi:10.1371/journal.pgen.1005773)
Supplement: S4 Fig — (A) Spectral evaluation of R19S in the range of NaOCl concentrations from 0.1 to 100 ppm. Left, A representative NaOCl dose-dependent change of R19S fluorescence. Right, The averaged graph of R19S fluorescence at indicated NaOCl concentrations (n = 4). (B) Typical confocal images of intestines with R19S. Experimental conditions were indicated at the left side of images. A part of the anterior midgut indicated as a red box in (C) is shown for each experiment. Field of view: 420 microns. (C) An R19S image of the midgut and hindgut from a fly that ingested uracil. R19S fluorescence was observed throughout the intestine, suggesting that HOCl production is not spatially limited. The red box indicates approximate location of the part shown in (B). (D) Ingestion of the R19S and 0.1 ppm NaOCl premixture yields the intensity and pattern of R19S fluorescence similar to the guts from animals that ingested 20 nM uracil. Right, A composite image illustrating the fluorescence distribution in the mid- and hindgut. (E) Averaged data are presented as bar graphs. Letters indicate significantly distinct groups. ANOVA Tukey, p<0.001. The number of experiments is indicated at the base of the graphs in grey. (PDF) [file pgen.1005773.s004.pdf]

Figure S4

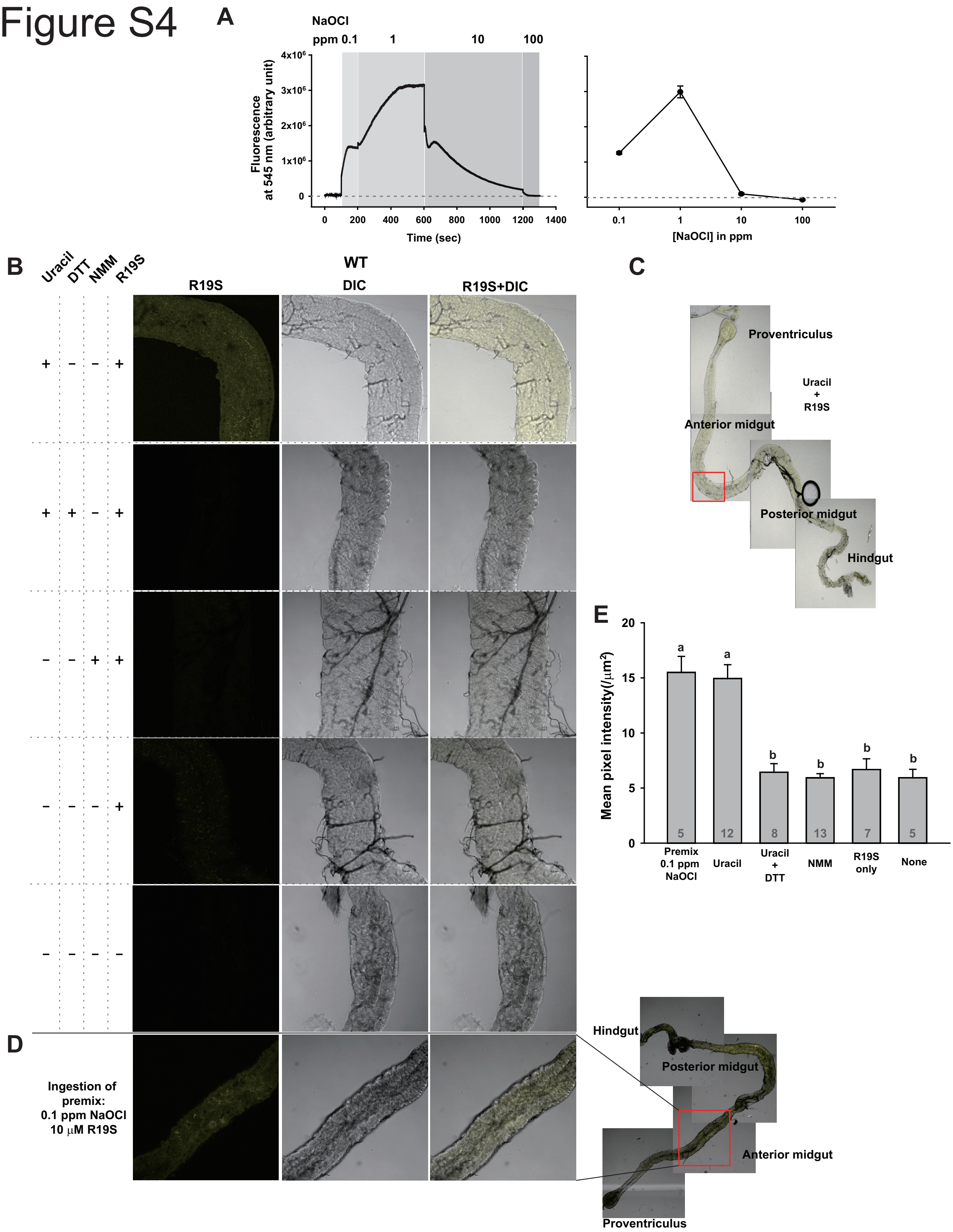

A
NaOCl
ppm 0.1
1
10
100
Fluorescence at 545 nm (arbitrary unit)
Time (sec)
[NaOCl] in ppm
B
Uracil
DTT
NMM
R19S
WT
R19S
DIC
R19S+DIC
C
Proventriculus
Uracil + R19S
Anterior midgut
Posterior midgut
Hindgut
E
Mean pixel intensity(/μm²)
Premix 0.1 ppm NaOCl
Uracil
Uracil + DTT
NMM
R19S only
None
D
Ingestion of premix: 0.1 ppm NaOCl 10 μM R19S
Hindgut
Posterior midgut
Anterior midgut
Proventriculus

Figure S4. The HOCl production in the gut was monitored with the use of the HOCl-specific dye R19S. (**A** Spectral evaluation of R19S in the range of NaOCl concentrations from 0.1 to 100 ppm. *Left*, A representative NaOCl dose-dependent change of R19S fluorescence. *Right*, The averaged graph of R19S fluorescence at indicated NaOCl concentrations (n=4). (**B**) Typical confocal images of intestines with R19S. Experimental conditions were indicated at the left side of images. A part of the anterior midgut indicated as a red box in (**C**) is shown for each experiment. Field of view: 420 microns. (**C**) An R19S image of the midgut and hindgut from a fly that ingested uracil. R19S fluorescence was observed throughout the intestine, suggesting that HOCl production is not spatially limited. The red box indicates approximate location of the part shown in (**B**). (**D**) Ingestion of the R19S and 0.1 ppm NaOCl premixture yields the intensity and pattern of R19S fluorescence similar to the guts from animals that ingested 20 nM uracil. *Right,* A composite image illustrating the fluorescence distribution in the mid- and hindgut. (**E**) Averaged data are presented as bar graphs. Letters indicate significantly distinct groups. ANOVA Tukey, $p<0.001$. The number of experiments is indicated at the base of the graphs in grey.
