# Supplementary material for: TrpA1 Regulates Defecation of Food-Borne Pathogens under the Control of the Duox Pathway
Source: PLoS Genet. 2016 Jan 4;12(1):e1005773. doi: 10.1371/journal.pgen.1005773 (PMC4699737; doi:10.1371/journal.pgen.1005773)
Supplement: S5 Fig — (A) Schematic diagram of the temporal control of Gal4/Gal80ts-dependent transcription. (B-D) Genetic interventions such as Duox (B) or TrpA1 (D) RNAi knockdown and Jafrac1 overexpression (C) by indicated Gal4 lines. Non-permissive temperature at 30°C disrupts transcriptional suppression of GAL80ts and allows GAL4 to drive transcription, while GAL80ts restricts transcription at permissive temperatures such as 18°C. **: p<0.01, ANOVA Tukey. (PDF) [file pgen.1005773.s005.pdf]

# Figure S5

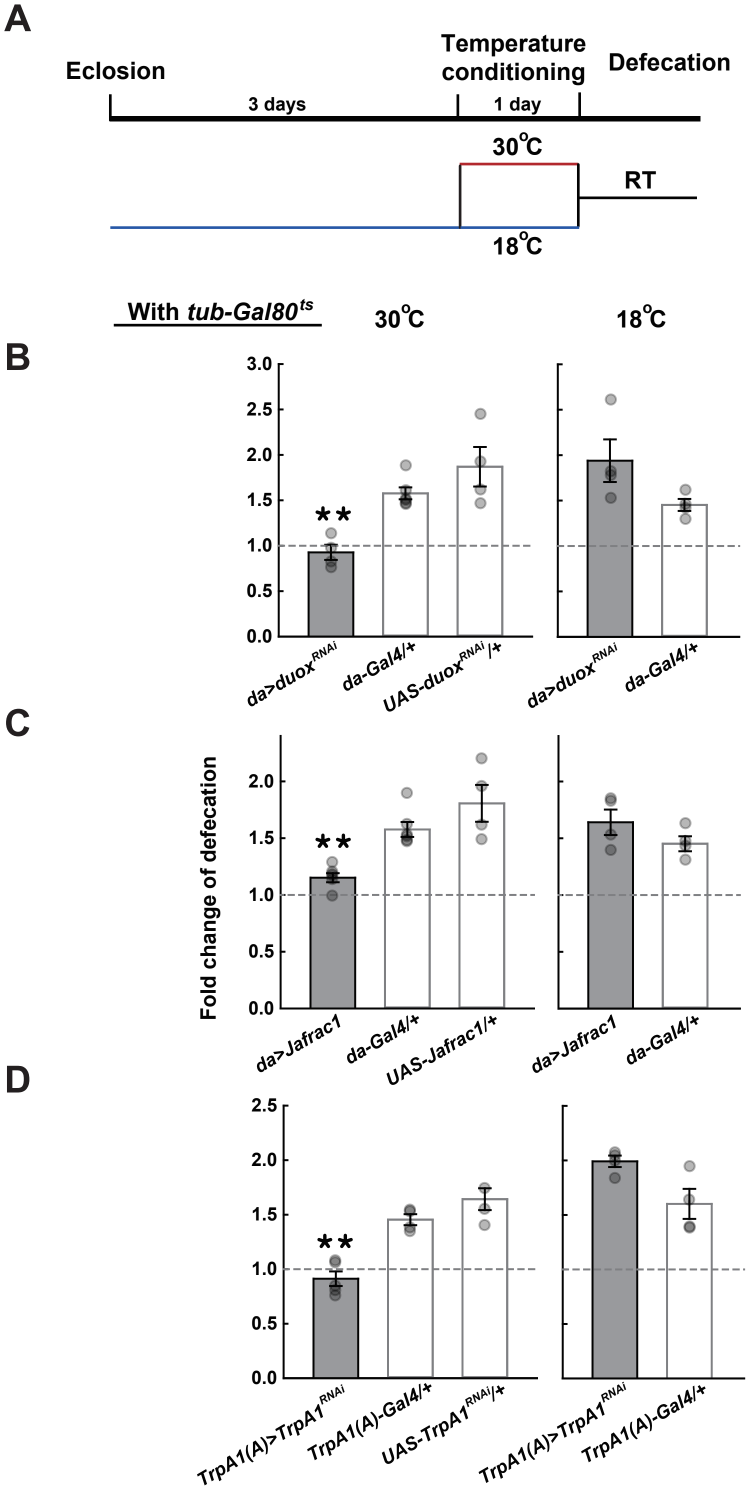


**Figure S5. Deficits in uracil-dependent defecation by genetic manipulations via the *Gal4/UAS* system are not due to developmental impairments.** (**A**) Schematic diagram of the temporal control of *Gal4/Gal80$^{ts}$*-dependent transcription. (**B**-**D**) Genetic interventions such as *Duox* (B) or *TrpA1* (D) RNAi knockdown and *Jafrac1* overexpression (C) by indicated *Gal4* lines. Non-permissive temperature at 30℃ disrupts transcriptional suppression of GAL80$^{ts}$ and allows GAL4 to drive transcription, while GAL80$^{ts}$ restricts transcription at permissive temperatures such as 18℃. **: p<0.01, ANOVA Tukey.
