# Supplementary figures and images for: TrpA1 Regulates Defecation of Food-Borne Pathogens under the Control of the Duox Pathway
Source: PLoS Genet. 2016 Jan 4;12(1):e1005773. doi: 10.1371/journal.pgen.1005773 (PMC4699737; doi:10.1371/journal.pgen.1005773)

Figure S6

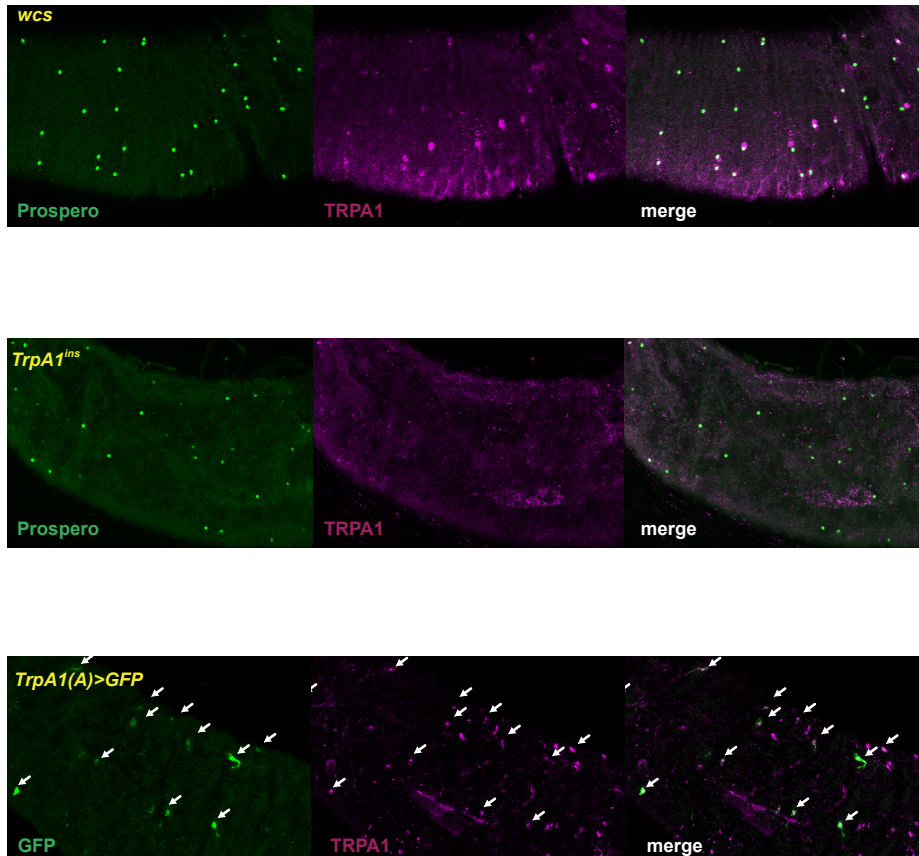

Figure S6. Higher magnification images of Figure 2A.

Supplement: S6 Fig — (PDF) [file pgen.1005773.s006.pdf]
