# Supplementary material for: TrpA1 Regulates Defecation of Food-Borne Pathogens under the Control of the Duox Pathway
Source: PLoS Genet. 2016 Jan 4;12(1):e1005773. doi: 10.1371/journal.pgen.1005773 (PMC4699737; doi:10.1371/journal.pgen.1005773)
Supplement: S7 Fig — (A) Schematic illustration of the gut regions stained with anti-TRPA1 antibody. (B) TrpA1(A)-Gal4 cells are enteroendocrine cells marked with anti-Prospero, except for large cells in the middle midgut that appear to be enterocytes (not shown). (C) TrpA1 RNAi knockdown in TrpA1(A)-Gal4 cells presented as “Output/Input” (n = 5–8). (PDF) [file pgen.1005773.s007.pdf]

Figure S7.

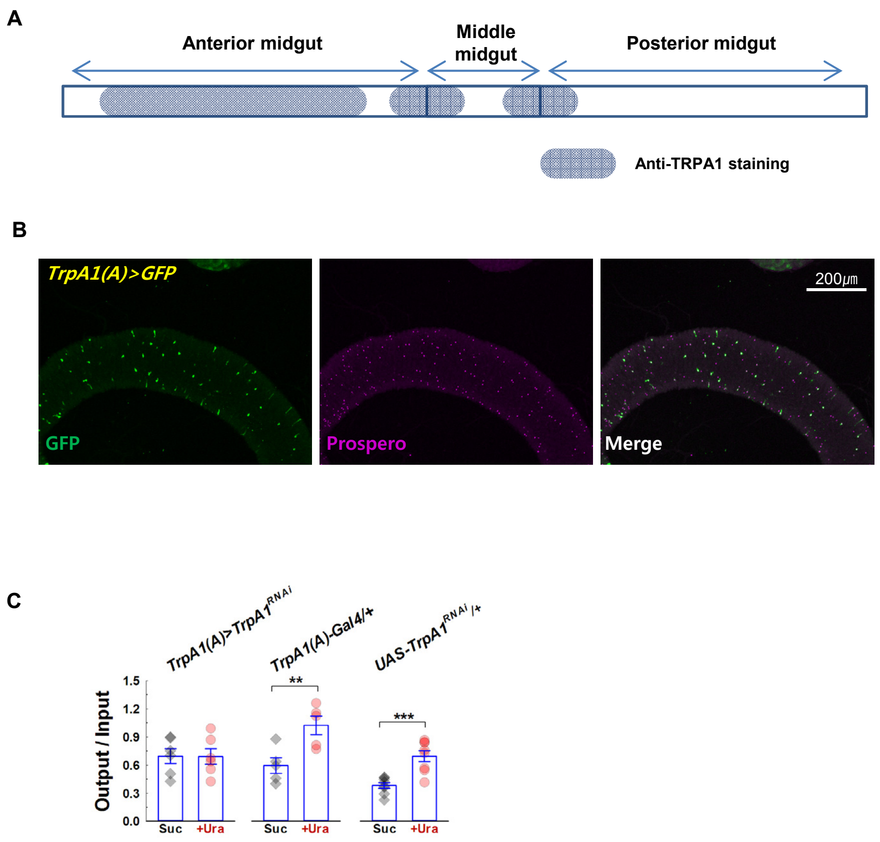

Figure S7. TRPA1 is expressed in enteroendocrine cells important for uracil-dependent defecation. (**A**) Schematic illustration of the gut regions stained with anti-TRPA1 antibody. (**B**) *TrpA1(A)-Gal4* cells are enteroendocrine cells marked with anti-Prospero, except for large cells in the middle midgut that appear to be enterocytes (not shown). (**C**) *TrpA1* RNAi knockdown in *TrpA1(A)-Gal4* cells presented as “Output/Input” (n=5-8).
