# Supplementary material for: TrpA1 Regulates Defecation of Food-Borne Pathogens under the Control of the Duox Pathway
Source: PLoS Genet. 2016 Jan 4;12(1):e1005773. doi: 10.1371/journal.pgen.1005773 (PMC4699737; doi:10.1371/journal.pgen.1005773)
Supplement: S8 Fig — TRPA1 staining results for RNAi knockdowned animals are summarized in the table (C). (PDF) [file pgen.1005773.s008.pdf]

# Figure S8

A

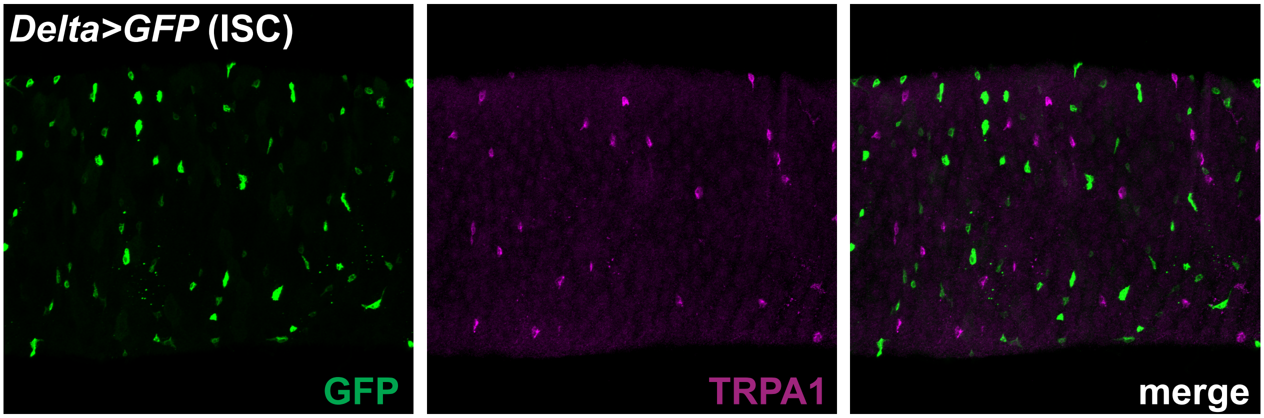


B

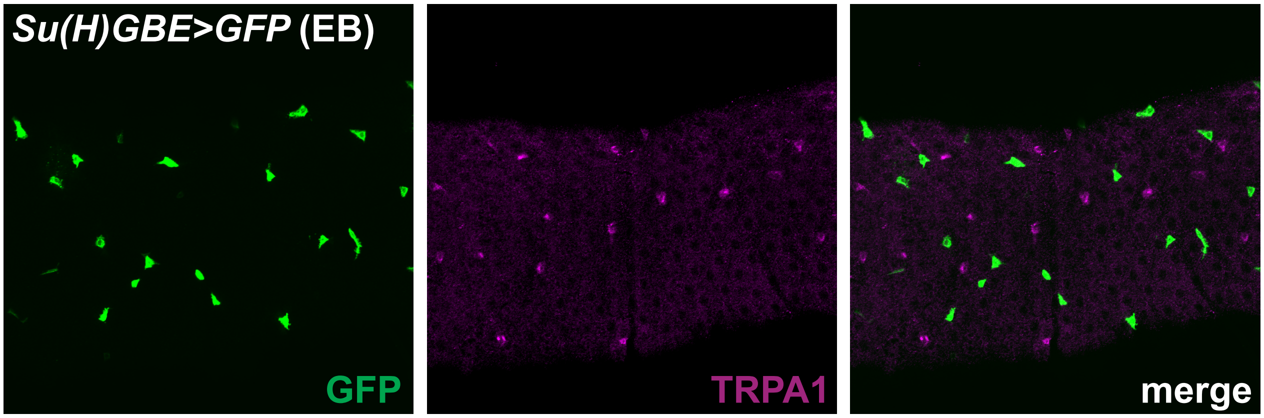


C

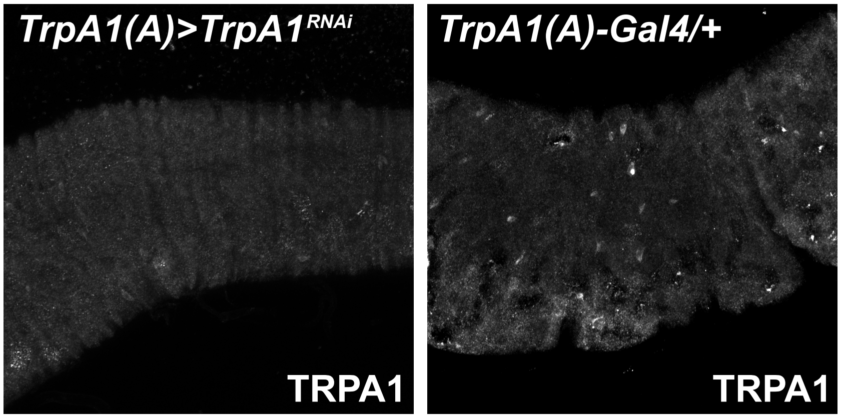


| EEC Staining | *TrpA1(A)> TrpA1$^{RNAi}$* | *TrpA1(A)-Gal4/+* | *UAS-TrpA1$^{RNAi}$ /+* |
|---|---|---|---|
| + | 0 | 8 | 7 |
| - | 5 | 1 | 2 |

p<0.01, Freeman-Halton extension of the Fisher's exact test

**Figure S8. Anti-TRPA1 staining reveals TRPA1-positive EECs not associated with intestinal stem cell (A) and enteroblast markers (B) and the *TrpA1* RNAi knockdown effect (C).** TRPA1 staining results for RNAi knockdowned animals are summarized in the table (**C**).
