# Supplementary material for: TrpA1 Regulates Defecation of Food-Borne Pathogens under the Control of the Duox Pathway
Source: PLoS Genet. 2016 Jan 4;12(1):e1005773. doi: 10.1371/journal.pgen.1005773 (PMC4699737; doi:10.1371/journal.pgen.1005773)
Supplement: S9 Fig — (A-B) Dose dependence (Left) and current voltage relationship (Right) of the temperature-sensitive Drosophila TRPA1 isoform, TRPA1(B)10a (A), and human TRPA1 (hsTRPA1, panel B). (C) Summarized dose dependence of hsTRPA1 (n = 4–10) and TRPA1(B)10a (n = 4–6). hsTRPA1 has a dose dependence profile very similar to TRPA1(B)10a. (D) Time constants from dose dependence experiments reveal that hsTRPA1 exhibits faster activation than TRPA1(B)10a but slower activation thanTRPA1(A) at 10 ppm NaOCl (n = 4–10). (E) Time constants of Drosophila TRPA1 isoforms and human TRPA1 (hsTRPA1) at -60 (Upper) and +60 mV (Lower) (n = 4–9). Shaded boxes indicate the concentrations that failed to generate sufficient currents for time constant determination. Note that only TRPA1(A)10b had time constants measured for 0.1 ppm. (F) EC50s from various TRPA1s at -60 and +60 mV (n = 4–10). EC50 of hsTRPA1 is comparable to that measured by a previous report [18]. *: p<0.05, ***: p<0.001, Tukey test vs. hsTRPA1 (D). ND: not determined. (G) Current amplitudes evoked by 100 ppm HOCl for estimation of expression in oocytes (n = 4–13). (H) L-bristles of animals with the indicated genotypes were examined for their NaOCl responsiveness. Note that Gr5a>TrpA1(A)10a taste neurons hardly showed action potentials to NaOCl 100 ppm, while responding to the immediately following NMM contact. In contrast, Gr5a cells expressing TRPA1(A)10b showed robust spikes from both sweet and water cells. The noisy low level spikes frequently appeared when the sensilla contact with NaOCl, probably due to its reactivity. (I) Summary of 30 sec-averaged spike frequencies evoked by NaOCl in L-bristles of flies used in (H) (n = 4–8). ***: p≤0.001, Tukey test. (PDF) [file pgen.1005773.s009.pdf]

Figure S9

A

B

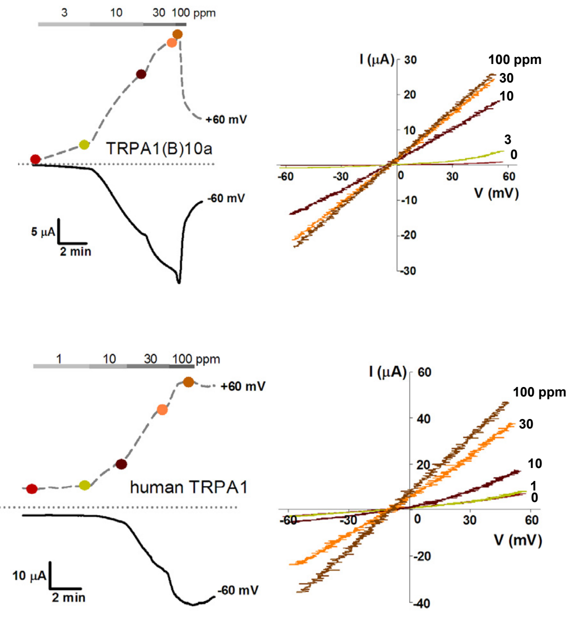

C

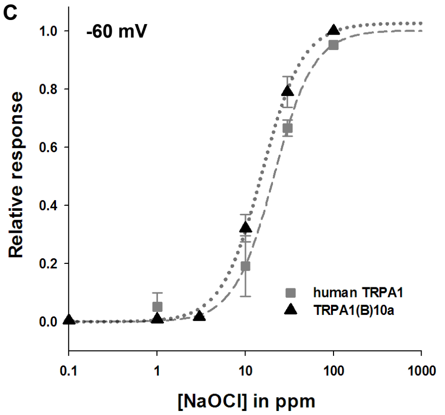

D

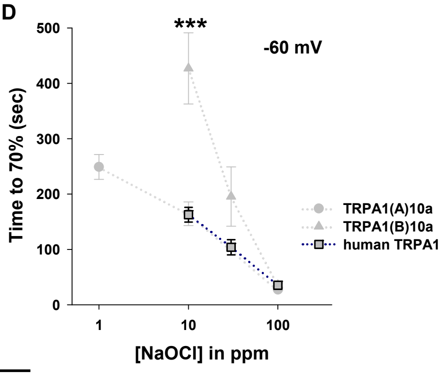

E

**Time to 70% (sec), -60 mV**	NaOCl concentration, ppm				
	0.1	1	10	30	100
TRPA1(A)10a	ND	249±22	165±21	ND	27±2
**TRPA1(A)10b**	131±10	78±6	17±2	ND	14±1
TRPA1(B)10a	ND	ND	369±62	178±50	22±3
hsTRPA1	ND	ND	131±16	53±9	22±2

**Time to 70% (sec), -60 mV**	NaOCl concentration, ppm				
	0.1	1	10	30	100
TRPA1(A)10a	ND	211±13	154±26	ND	15±2
**TRPA1(A)10b**	138±11	78±5	15±1	ND	13±1
TRPA1(B)10a	ND	ND	369±62	179±50	22±3
hsTRPA1	ND	ND	164±14	57±8	30±3

F

**EC50s (ppm)**	-60 mV	+60 mV
TRPA1(A)10a	5.7±0.6	3.7±0.4
**TRPA1(A)10b**	1.7±0.7	0.7±0.2
TRPA1(B)10a	13.2±1.0	8.7±0.4
hsTRPA1	21.1±3.6	19.3±5.6

G

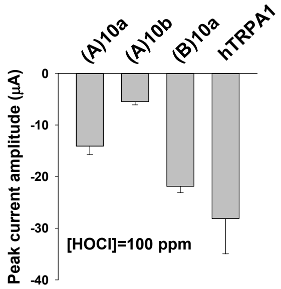

Figure S9. Continued to the next page.

Figure S9 (continued)

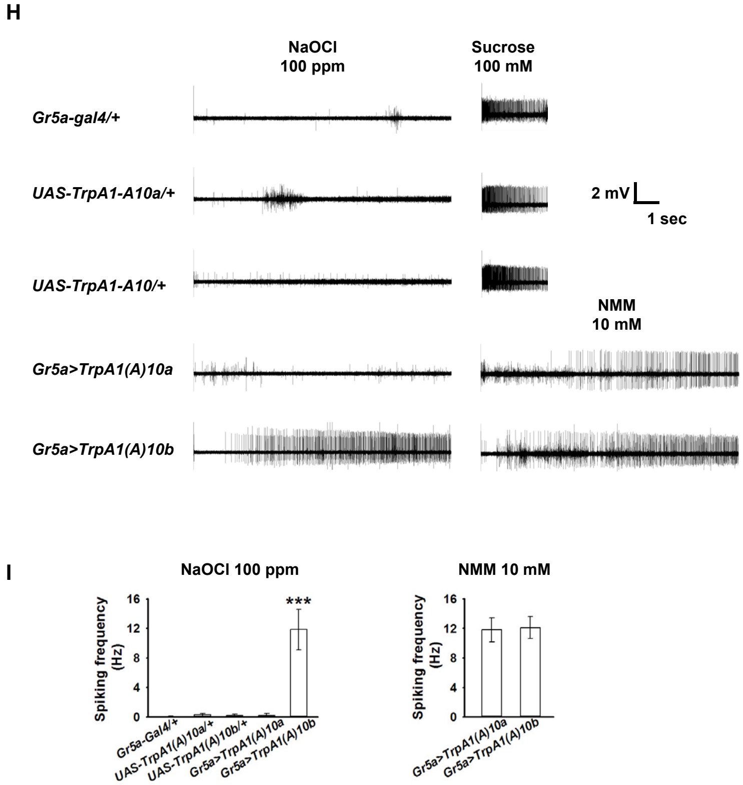

Figure S9. NaOCl response parameters of *Drosophila* TRPA1 isoforms and human TRPA1 heterologously expressed in oocytes, and differential HOCl responses of TRPA1 isoforms ectopically expressed in sugar-sensing *Gr5a* cells. (**A**-**B**) Dose dependence (*Left)* and current-voltage relationship (*Right*) of the temperature-sensitive *Drosophila* TRPA1 isoform, TRPA1(B)10a (**A**), and human TRPA1 (hsTRPA1, panel **B**). (**C**) Summarized dose dependence of hsTRPA1 (n=4-10) and TRPA1(B)10a (n=4-6). hsTRPA1 has a dose dependence profile very similar to TRPA1(B)10a. (**D**) Time constants from dose dependence experiments reveal that hsTRPA1 exhibits faster activation than TRPA1(B)10a but slower activation thanTRPA1(A) at 10 ppm NaOCl (n=4-10). (**E**) Time constants of *Drosophila* TRPA1 isoforms and human TRPA1 (hsTRPA1) at -60 (*Upper*) and +60 mV (*Lower*) (n=4-9). Shaded boxes indicate the concentrations that failed to generate sufficient currents for time constant determination. Note that only TRPA1(A)10b had time constants measured for 0.1 ppm. (**F**) EC50s from various TRPA1s at -60 and +60 mV (n=4-10). EC50 of hsTRPA1 is comparable to that measured by a previous report [18]. *: $p<0.05$, ***: $p<0.001$, Tukey test vs. hsTRPA1 (**D**). ND: not determined. (**G**) Current amplitudes evoked by 100 ppm HOCl for estimation of expression in oocytes (n=4-13). (**H**) L-bristles of animals with the indicated genotypes were examined for their NaOCl responsiveness. Note that *Gr5a>TrpA1(A)10a* taste neurons hardly showed action potentials to NaOCl 100 ppm, while responding to the immediately following NMM contact . In contrast, *Gr5a* cells expressing TRPA1(A)10b showed robust spikes from both sweet and water cells. The noisy low level spikes frequently appeared when the sensilla contact with NaOCl, probably due to its reactivity. (**I**) Summary of 30 sec-averaged spike frequencies evoked by NaOCl in L-bristles of flies used in (**H**) (n=4-8). ***: $p\leq0.001$, Tukey test.
