# Supplementary material for: TrpA1 Regulates Defecation of Food-Borne Pathogens under the Control of the Duox Pathway
Source: PLoS Genet. 2016 Jan 4;12(1):e1005773. doi: 10.1371/journal.pgen.1005773 (PMC4699737; doi:10.1371/journal.pgen.1005773)
Supplement: S10 Fig — The non-covalent agonist citronellal opens TRPA1(A)10b faster and more sensitively than TRPA1(A)10a. Typical current recording with increasing concentrations of citronellal for TRPA1(A)10a (A) and TRPA1(A)10b (B). (C) Citronellal dose dependences of the two isoforms. (D) Time to 70% amplitude at each concentration was plotted as function of citronellal concentrations. (PDF) [file pgen.1005773.s010.pdf]

Figure S10

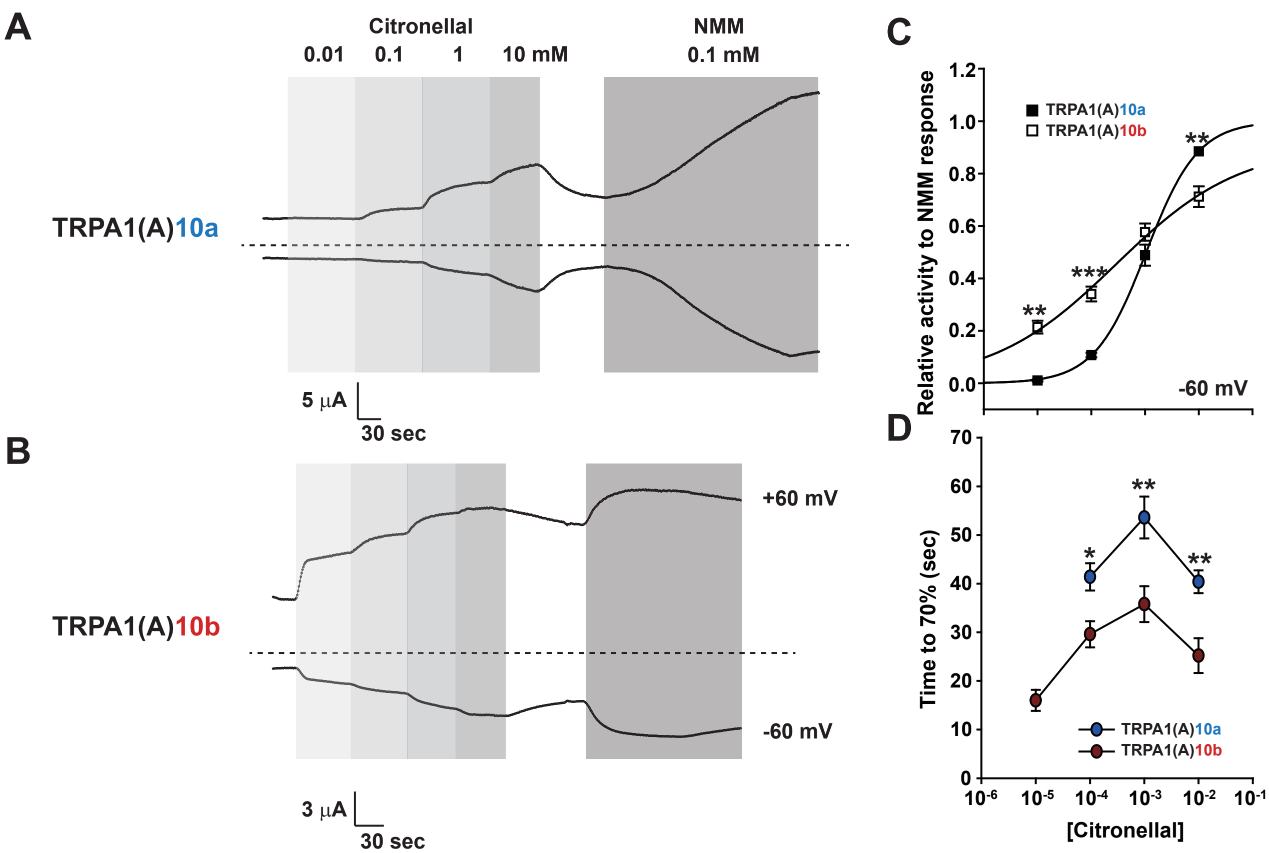

Figure S10. **TRPA1(A)10b responds fast and sensitively to citronellal compared to TRPA1(A)10a, when expressed in frog oocytes.** The non-covalent agonist citronellal opens TRPA1(A)10b faster and more sensitively than TRPA1(A)10a. Typical current recording with increasing concentrations of citronellal for TRPA1(A)10a (**A**) and TRPA1(A)10b (**B**). (**C**) Citronellal dose dependences of the two isoforms. (D) Time to 70% amplitude at each concentration was plotted as function of citronellal concentrations.
