# Supplementary material for: TrpA1 Regulates Defecation of Food-Borne Pathogens under the Control of the Duox Pathway
Source: PLoS Genet. 2016 Jan 4;12(1):e1005773. doi: 10.1371/journal.pgen.1005773 (PMC4699737; doi:10.1371/journal.pgen.1005773)
Supplement: S11 Fig — Upper, Output/input results were presented for indicated genotypes. Defecation frequencies were normalized to the feeding amounts after ingestion of either ECC15 WT or pyrE. Lower, The results in the Upper panel were displayed as fold change of defecation for estimation of fold increase of defecation by uracil from ECC15. **: p<0.01, ANOVA Tukey or Student t-test. (PDF) [file pgen.1005773.s011.pdf]

Figure S11

Germ-free

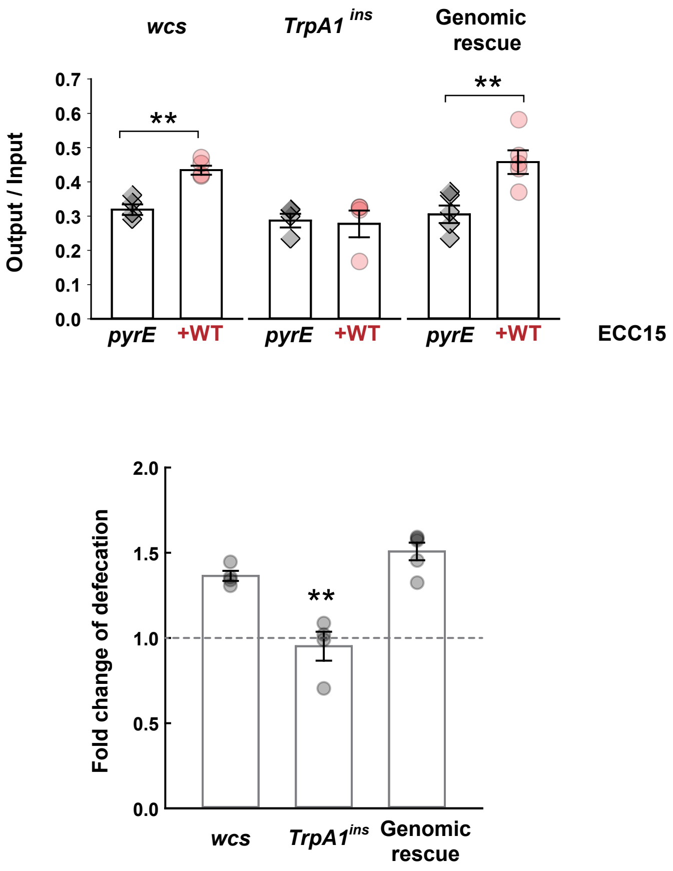

Figure S11. ECC15-dependent defecation in germ-free *Drosophila melanogaster.* *Upper,* Output/input results were presented for indicated genotypes. Defecation frequencies were normalized to the feeding amounts after ingestion of either ECC15 WT or *pyrE*. *Lower,* The results in the *Upper* panel were displayed as fold change of defecation for estimation of fold increase of defecation by uracil from ECC15. **: $p<0.01$, ANOVA Tukey or Student *t*-test.
