# Supplementary material for: TrpA1 Regulates Defecation of Food-Borne Pathogens under the Control of the Duox Pathway
Source: PLoS Genet. 2016 Jan 4;12(1):e1005773. doi: 10.1371/journal.pgen.1005773 (PMC4699737; doi:10.1371/journal.pgen.1005773)
Supplement: S12 Fig — (A) NaOCl resistance was assessed with ECC15-GFP that previously resided in wcs or TrpA1ins guts in the range of indicated concentrations and is not significantly different between the two genotypes. (B) ECC15-GFP from TrpA1ins guts opportunistically survive the NaOCl concentration that eliminated the growth of the bacteria from wcs guts. (C) Enhanced survival to ingested ECC15 pyrE of TrpA1ins is not rescued by reintroduction of the TrpA1 genomic DNA, suggesting that the resistance TrpA1ins exhibited might have originated from its genetic background. (PDF) [file pgen.1005773.s012.pdf]

Figure S12

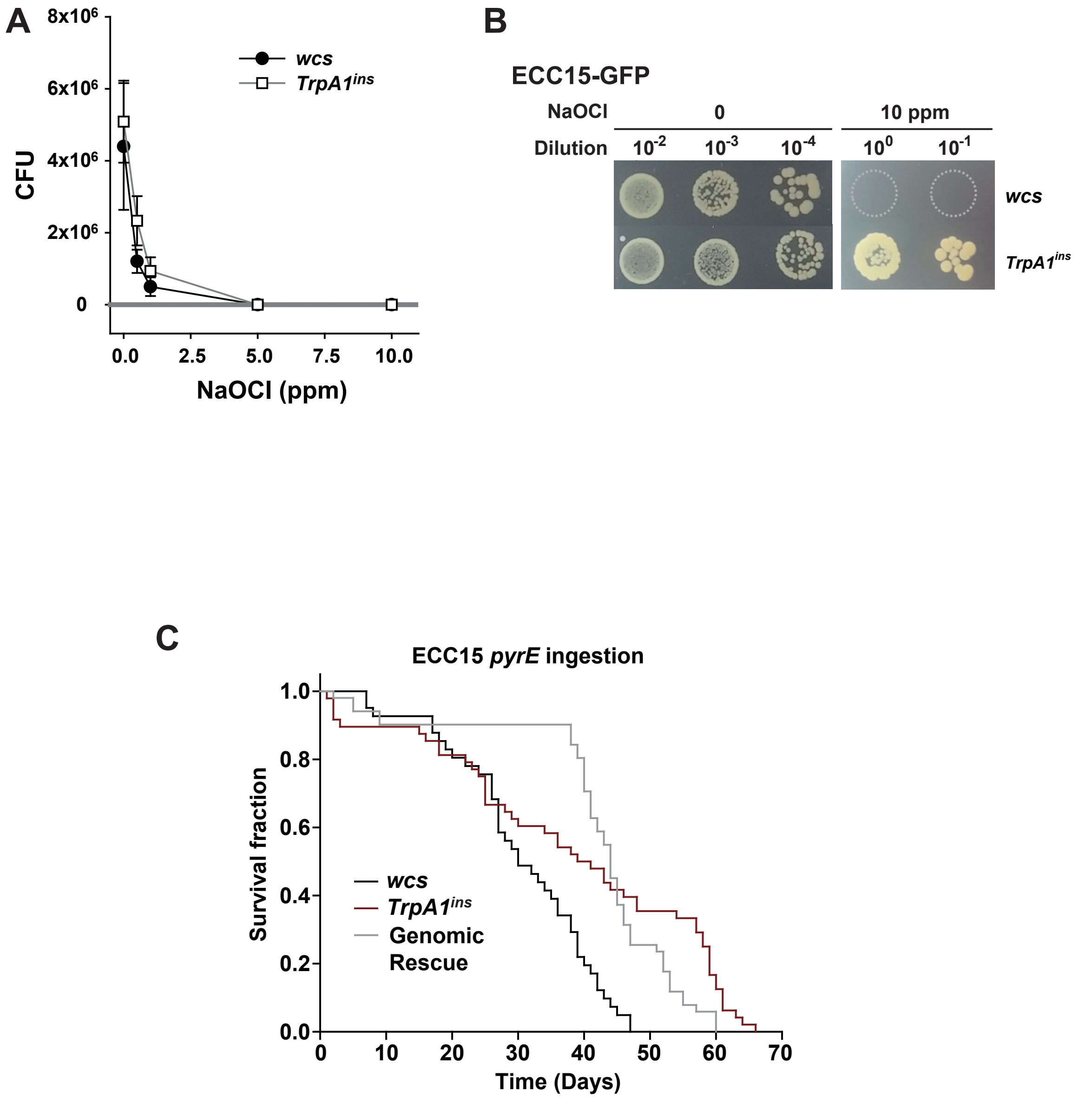

Figure S12. Transiently acquired NaOCl resistance of ECC15 from the *TrpA1^{ins}* gut and mortality rates of flies upon ingestion of ECC15 *pyrE*. (**A**) NaOCl resistance was assessed with ECC15-GFP that previously resided in *wcs* or *TrpA1^{ins}* guts in the range of indicated concentrations and is not significantly different between the two genotypes. (**B**) ECC15-GFP from *TrpA1^{ins}* guts opportunistically survive the NaOCl concentration that eliminated the growth of the bacteria from *wcs* guts. (**C**) Enhanced survival to ingested ECC15 *pyrE* of *TrpA1^{ins}* is not rescued by reintroduction of the *TrpA1* genomic DNA, suggesting that the resistance *TrpA1^{ins}* exhibited might have originated from its genetic background.
